# Eps8 Regulates Hair Bundle Length and Functional Maturation of Mammalian Auditory Hair Cells

**DOI:** 10.1371/journal.pbio.1001048

**Published:** 2011-04-19

**Authors:** Valeria Zampini, Lukas Rüttiger, Stuart L. Johnson, Christoph Franz, David N. Furness, Jörg Waldhaus, Hao Xiong, Carole M. Hackney, Matthew C. Holley, Nina Offenhauser, Pier Paolo Di Fiore, Marlies Knipper, Sergio Masetto, Walter Marcotti

**Affiliations:** 1 Department of Biomedical Science, University of Sheffield, Sheffield, United Kingdom; 2 Department of Physiology, University of Pavia, Pavia, Italy; 3 Department of Otolaryngology, THR, University of Tübingen, Tübingen, Germany; 4 Institute for Science and Technology in Medicine, Keele University, Keele, United Kingdom; 5 Department of Otolaryngology-Head & Neck Surgery, Tongji Hospital, Tongji Medical College, Huazhong University of Science & Technology, Wuhan, China; 6 IFOM, Fondazione Istituto FIRC di Oncologia Molecolare, Milan, Italy; 7 Istituto Europeo di Oncologia, Milan, Italy; 8 Dipartimento di Medicina, Chirurgia e Odontoiatria, Università degli Studi di Milano, Milan, Italy; Baylor College of Medicine, United States of America

## Abstract

Hair cells of the mammalian cochlea are specialized for the dynamic coding of sound stimuli. The transduction of sound waves into electrical signals depends upon mechanosensitive hair bundles that project from the cell's apical surface. Each stereocilium within a hair bundle is composed of uniformly polarized and tightly packed actin filaments. Several stereociliary proteins have been shown to be associated with hair bundle development and function and are known to cause deafness in mice and humans when mutated. The growth of the stereociliar actin core is dynamically regulated at the actin filament barbed ends in the stereociliary tip. We show that Eps8, a protein with actin binding, bundling, and barbed-end capping activities in other systems, is a novel component of the hair bundle. Eps8 is localized predominantly at the tip of the stereocilia and is essential for their normal elongation and function. Moreover, we have found that Eps8 knockout mice are profoundly deaf and that IHCs, but not OHCs, fail to mature into fully functional sensory receptors. We propose that Eps8 directly regulates stereocilia growth in hair cells and also plays a crucial role in the physiological maturation of mammalian cochlear IHCs. Together, our results indicate that Eps8 is critical in coordinating the development and functionality of mammalian auditory hair cells.

## Author Summary

Mammalian hearing depends on mechanosensory inner and outer hair cells within the inner ear that convert sound vibrations into electrical signals. While inner hair cells are the primary sensory receptors, outer hair cells improve auditory sensitivity. Although we know that sensory cells of the auditory, visual and olfactory systems undergo a series of regulated physiological and morphological changes during development, relatively little is known about the molecular mechanisms that regulate the development of these cells. In this study, we find that the protein Eps8, which binds to the key structural protein actin and regulates cell growth and neural development, is an essential component of auditory hair cell development and function. We show that mice lacking Eps8 are profoundly deaf and that their mechanically sensitive hair bundles do not fully grow. However, we also show that the bundles retain their ability to transduce mechanical stimuli. Further study revealed that Eps8 has additional functions in the physiological maturation of inner hair cells and in their ability to transmit electrical information to the brain. Combined, our results provide evidence for the complex physiological role of Eps8 in hair cells and the reason why its absence causes profound deafness.

## Introduction

The mechanoelectrical transduction of sound information is made possible by sensory hair cells (inner and outer hair cells) in the cochlea [1]. The initial step in the sound transduction cascade is performed by mechanically gated ion channels positioned near the tips of hair cell stereocilia. Stereocilia are microvilli-like structures that protrude from the apical surface of hair cells, with a core composed of tightly packed actin filaments [1],[2]. Their lengths are scaled precisely to form bundles of stereocilia (hair bundle) with a staircase-like architecture [3],[4]. Each hair bundle is composed of two or more rows of stereocilia that are coupled to one another by extracellular links of several types [2]. The staircase mainly develops postnatally when stereociliary elongation stops initially in the shortest rows at around postnatal day 5 (P5) and the tallest row at about P15 in mice [3]. The height of stereocilia within a row is similar not only within a single hair bundle but also in the bundles of closely adjacent hair cells, indicating that the polymerization and depolymerization of their F-actin core is tightly regulated [4].

Several genes encoding for stereociliary proteins, including whirlin [5],[6], espin [7],[8], and the unconventional myosins VIIa [9] and XVa [10], have been shown to cause deafness when mutated [2]. Although these proteins are important for the correct regulation of hair bundle length and development, they are unlikely to control actin polymerization directly [2]. Recently, it has been shown that the novel stereociliary protein twinfilin 2, an actin filament barbed-end capping protein located only at the tips of the short and middle rows of stereocilia in IHCs, is able to control actin dynamics in developing and mature hair bundles by restricting their excessive elongation [3]. However, the nature of the protein(s) regulating actin dynamics in the tallest stereocilia remains unknown. Epidermal growth factor receptor pathway substrate 8 (Eps8 [11],[12]) is an evolutionarily conserved signal transducer endowed with multiple functions in the control of actin dynamics and in the integration of these events with other receptor-activated signaling functions. Depending on its association with other signal transducers, Eps8 can regulate the activation of the Rac GTPase, a master regulator of actin remodeling [13]–[15], and integrate cellular signaling and membrane EGF receptor internalization [16]. Eps8 can also act directly on actin by binding to it and exerting both actin bundling and actin barbed-end capping activity [14],[17],[18]. At the functional level, Eps8 has been implicated in axonal filopodia growth [19], in modulating the activity of the NMDA receptor [20], and in regulating the length of intestinal microvilli, which were found to be shorter in its absence [17],[21]. Given the structural similarity between microvilli and stereocilia, we hypothesized that Eps8 may also be important for hair cell stereocilia growth and more generally to hair-cell function. To test these hypotheses we undertook a structural and an in-depth physiological investigation of Eps8 knockout mice. We report that hair cells from these mice indeed exhibit shorter stereocilia. Moreover, Eps8 knockout mice are deaf and the normal physiological development of IHCs is prevented. Thus our results identify a novel critical regulator of one of the most distinctive functional refinements of the mammalian auditory system.

## Results

### Eps8 Is Present at the Tips of the Hair Cell Stereocilia

**Figure 1:**
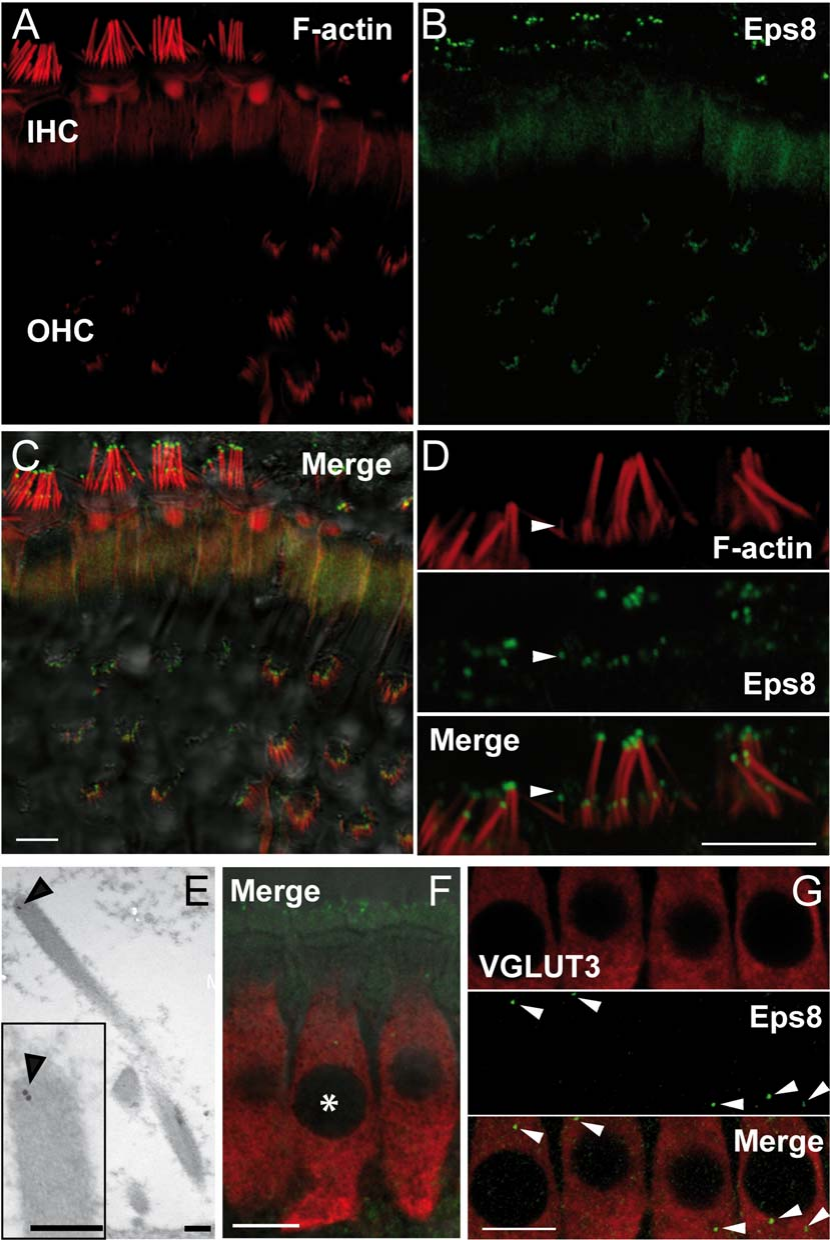
Eps8 is localized at the hair cells stereociliary tips. (A–C) F-actin containing stereocilia (A) and Eps8 (B) staining in IHCs and OHCs from P45 mice using confocal microscopy. Merged images are shown in (C) together with the DIC image. Note that Eps8 localizes at the tip of stereocilia (red: F-actin staining). (D) High resolution image of IHC stereocilia showing that Eps8 is present in the tips of both taller and shorter (arrowhead) stereocilia. Scale bars in (A–D) are 5 µm. (E) TEM immunogold labeling showing the stereociliary localization of Eps8 in a P35 IHC (inset shows this at higher magnification). Scale bars: 200 nm. (F) Immunostaining of IHCs from P16 mice using confocal microscopy. Image is a single section at the level of the IHC nuclei. Red indicates the IHC marker VGLUT3 and green Eps8. Asterisks denote IHC nuclei. Note that some stereociliary Eps8 staining is evident (top of the image) due to the angle of the tissue. (G) Magnified images of IHCs showing that punctate Eps8 labeling was also present in the cell cytoplasm (arrowheads). Scale bars in (F) and (G): 10 µm.

The localization of Eps8 in cochlear hair cells was determined by performing immunofluorescence and post embedding immunogold labeling on immature and adult wild-type control mice. Eps8 expression was detected at the tip of the stereocilia of cochlear hair cells (Figure 1A–D and Figure S1A–C). The specificity of the antibody was verified on hair cells from Eps8 knockout mice (Figure S1D–F). The expression pattern of Eps8 was also confirmed by immunogold labeling (Figure 1E, example for an adult IHCs), with a pattern remarkably reminiscent of the localization of Eps8 at the tip of intestinal microvilli [17]. Punctate expression of Eps8, similar to that observed in the stereocilia (Figure 1A–E), was also present in the IHC cytoplasm (Figure 1F,G) and was not observed in the absence of the primary antibody for Eps8 (unpublished data). This pattern of Eps8 expression was further confirmed by immunogold experiments where gold particles were observed in the cytoplasm of IHCs (density of 3 particles/µm^2^ measured from 10 IHCs) at higher levels than over the tissue-free resin of the section. These findings indicate that there is likely to be some expression of Eps8 in the cell cytoplasm.

### Hair Cells from Eps8 Knockout Mice Have Shorter Stereocilia

**Figure 2:**
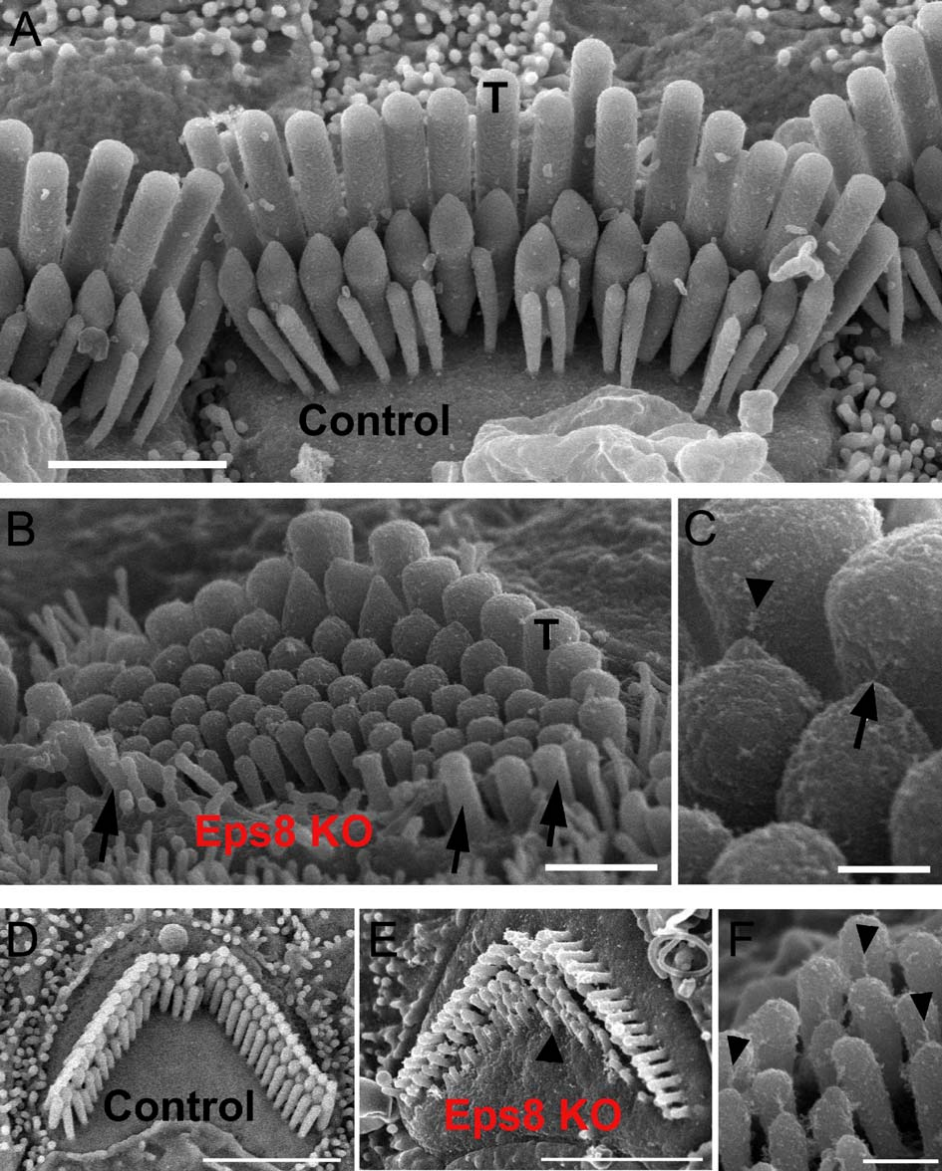
Hair bundle morphology in hair cells from adult Eps8 mice. (A) Scanning electron micrograph (SEM) showing the typical hair bundle structure in control adult mouse P18 IHCs. Note that, generally, IHC hair bundles are composed of three rows of stereocilia. The tall stereocilia are indicated by T. Scale bar: 2 µm. (B) SEM showing that tall stereocilia (T) appear to be truncated in adult Eps8 knockout mice. Arrows indicate taller stereocilia oriented opposite to those positioned in the direction of bundle sensitivity. Moreover, additional rows of short stereocilia (three in this case) are present compared to wild type mice. Scale bar: 1 µm. (C) Higher magnification micrograph showing a tip link (arrow) and tip-link remnant (arrowhead) in an IHC from an Eps8 knockout mouse. Scale bar: 250 nm. (D–F) Outer hair cell hair bundle morphology in Eps8 mice. SEM showing the typical hair bundle structure of a second row (from the modiolus) OHC from the middle region of a normal wild type adult mouse cochlea (panel D). The precisely arranged rows of stereocilia with well defined height increments can be seen. (E) A comparable hair bundle from an Eps8 adult mutant mouse. Note that all the stereocilia in equivalent rows appear shorter, and the shortest row has missing stereocilia. Additional rows of stereocilia are also present (arrowhead). Scale bars: 2.5 µm. (F) Detail of the hair bundle of an OHC from row 1 showing numerous tip links including some that are single filaments (arrowheads). Scale bar: 300 nm.

**Figure 3:**
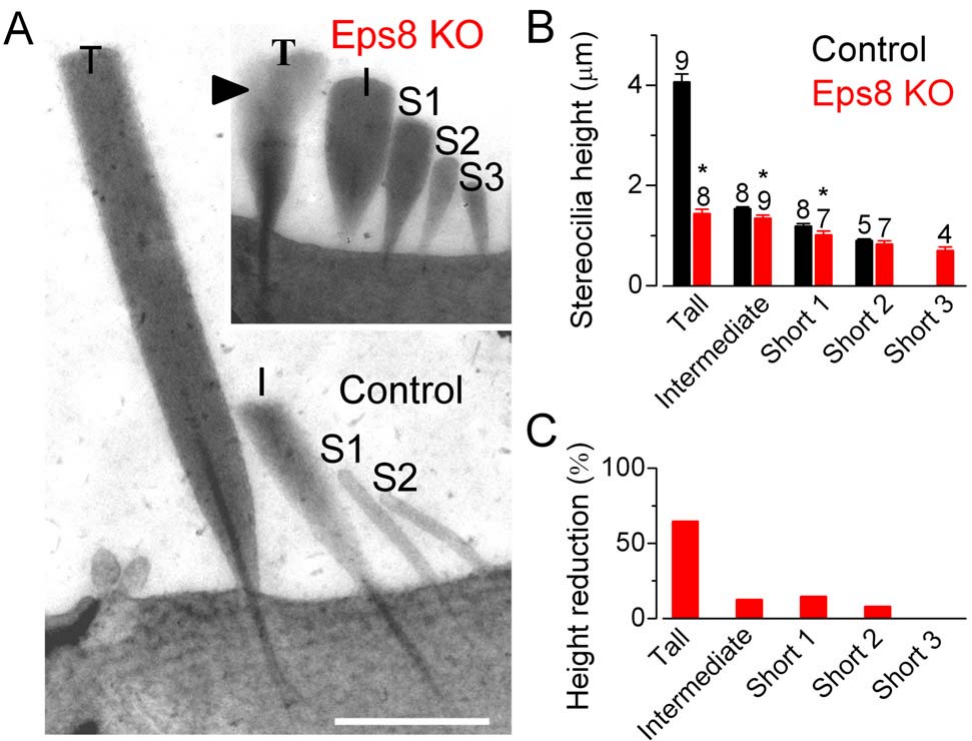
IHC hair bundle height in adult Eps8 mice. (A) Transmission electron micrographs (TEM) of apical-coil IHCs from wild type (control) and Eps8 knockout (KO) adult mice (P22–P35) showing hair bundles in radial semi-thin sections. Semi-thin sections were used to maximize stereocilium length within a single section but do not allow clear visualization of finer structures such as tip links. Note the four rows of stereocilia in the control labeled: tall (T), intermediate (I), and two shorter rows (S1, S2). The knockout hair bundle is shown at the same magnification in the inset. Equivalent rows (T, I, S1, and S2) are identified by location across the hair bundle; an additional short row (S3) is also present. In the knockout hair bundle, portions of two overlapping tall stereocilia are visible (arrowhead); note that the tips (e.g. height) of these two overlapping stereocilia were similar. Scale bars: 1 µm. (B) Heights of stereocilia (mean ± s.d., number of stereocilia measured from 3 knockout and 4 control IHCs is shown above the bars). Asterisks indicate statistical significance. (C) Reduction (%) in stereocilia height in knockout IHCs compared to those of control cells.

Scanning (SEM: Figure 2A–C for IHCs and Figure 2D–F for OHCs) and transmission electron microscopy (TEM: Figure 3 for IHCs) were used to investigate the hair bundle morphology in Eps8 knockout mice. IHC stereocilia from Eps8 knockout mice were shorter than those of wild-type animals (Figures 2 and 3). Hair bundles of IHCs from Eps8 knockouts also had additional rows of stereocilia (five or six instead of the typical three to four rows: Figure 2A,B), resembling those of immature hair cells [22]. OHCs from knockout mice also had shorter and additional rows of stereocilia compared to control cells (Figure 2D,E). Despite the defects associated with the absence of Eps8, the staircase-like architecture of hair bundles [4] was preserved, although shallower (Figures 2 and 3), and tip links were present in both immature and adult knockout hair cells (Figure 2C and F for an adult IHC and OHC, respectively). We measured the lengths of individual stereocilia in IHCs from TEM images. We found that in knockout IHCs the first three rows of stereocilia were significantly shorter when compared to controls (Figure 3). The height of tall stereocilia (Figure 3B) was 4.1±0.2 µm (*n* = 9) in control compared with 1.4±0.1 µm (*n* = 8) in knockout cells (*p*<0.0001) with an overall reduction of 65% (Figure 3C). The height of the intermediate (control: 1.5±0.1 µm, *n* = 8; knockout: 1.3±0.1 µm, *n* = 9, *p*<0.01) and first shortest row (Short 1, control: 1.2±0.1 µm, *n* = 8; knockout: 1.0±0.1 µm, *n* = 7, *p*<0.05) stereocilia were also found to be reduced by 13% and 15%, respectively, in knockout cells. The second shortest row (Short 2) stereociliary height did not change significantly between controls and knockout cells (control: 0.9±0.1 µm, *n* = 5; knockout: 0.8±0.1 µm, *n* = 7).

### Eps8 Knockout Mice Exhibit Profound Hearing Loss

**Figure 4:**
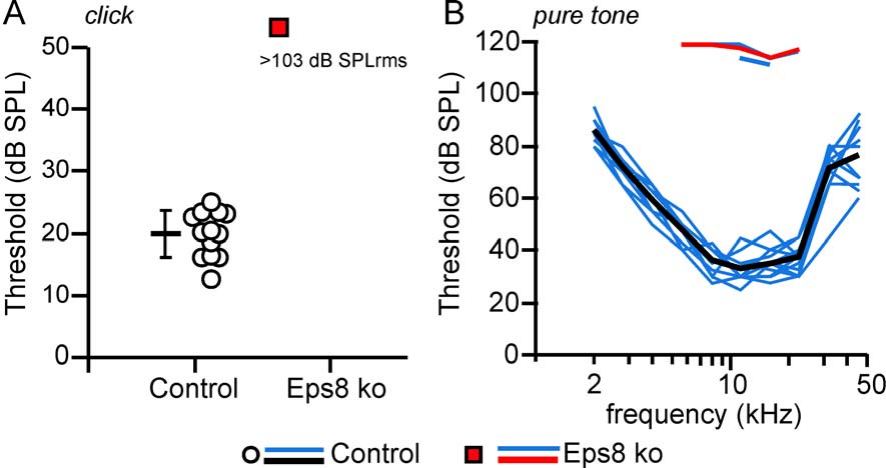
Auditory brainstem responses (ABR) in Eps8 mice. (A–B) ABR thresholds, obtained from control (13 ears from 7 mice: circles and black line) and Eps8 knockout (16 ears from 8 mice: red squares and red line) adult mice. ABR thresholds for broadband click (A) and frequency-specific pure tone stimulation (B) in Eps8 knockout mice were significantly higher than those in control mice (click and noise pulse *p*<0.001; pure tone *p*<0.001 between 5.6 and 22.3 kHz). Note that for frequency-specific ABR, threshold could only be determined in 3 ears from knockout mice (no thresholds were determined in all animals for click stimulation). Thresholds of individual ears are shown as circle and square symbols (click) and blue lines (pure tone). Black and red lines give the mean values. Responses to pure tone stimuli in knockout mice could only be recorded at high stimulus levels outside the normal physiological range (B: pure tone threshold 110–120 dB SPL).

The physiological consequences of the absence of Eps8 in hair cells were investigated by testing hearing function in Eps8 knockout adult mice (P30–P57) using auditory brainstem responses (ABRs) and electrocochleography. Eps8 knockouts were profoundly deaf since ABRs, which reflect the activity of the afferent auditory pathway and IHCs, could only be elicited in response to unphysiologically high sound stimulus levels (pure tone threshold in knockout mice was 110–120 dB SPL: Figure 4). ABR thresholds for broadband click (Figure 4A), noise pulse stimuli (unpublished data), and frequency-specific pure tone stimulation (Figure 4B) in Eps8 knockout mice were significantly higher than those in control mice (*p*<0.001 for click and noise pulse and pure tone). In order to investigate cochlear function in more detail we used electrocochleography (CAP, SP, and CM) and DPOAEs. Compound action potentials (CAPs), which represent the firing activity of auditory afferent fibres, were greatly reduced in Eps8 knockout mice compared to those in control animals (Figures S2). The thresholds for eliciting summating potentials (SPs: reflecting the summation of IHC depolarization) were also significantly higher in Eps8 knockout mice (Figures S3). The endocochlear potential (EP), which reflects the electrical driving force for mechanoelectrical transduction, was found to be similar between Eps8 knockout (81.3±6.5 mV) and control mice (85.1±5.3 in 10–11-wk-old mice), indicating that the driving force for generating the IHC receptor potential is likely to be normal.

The activity of OHCs was investigated by measuring distortion product otoacoustic emissions (DPOAEs) and cochlear microphonics (CM). In knockout animals both DPOAEs and CM thresholds were increased to stimulus levels close to the technical detection limit (Figure S4). The reduced physiological responses in knockout mice could be related to hair cell abnormalities such as in the mechanoelectrical transduction apparatus (stereocilia and transducer channels) and/or altered biophysical properties of their basolateral membrane.

### Hair Cell Transducer Current Does Not Require Eps8

**Figure 5:**
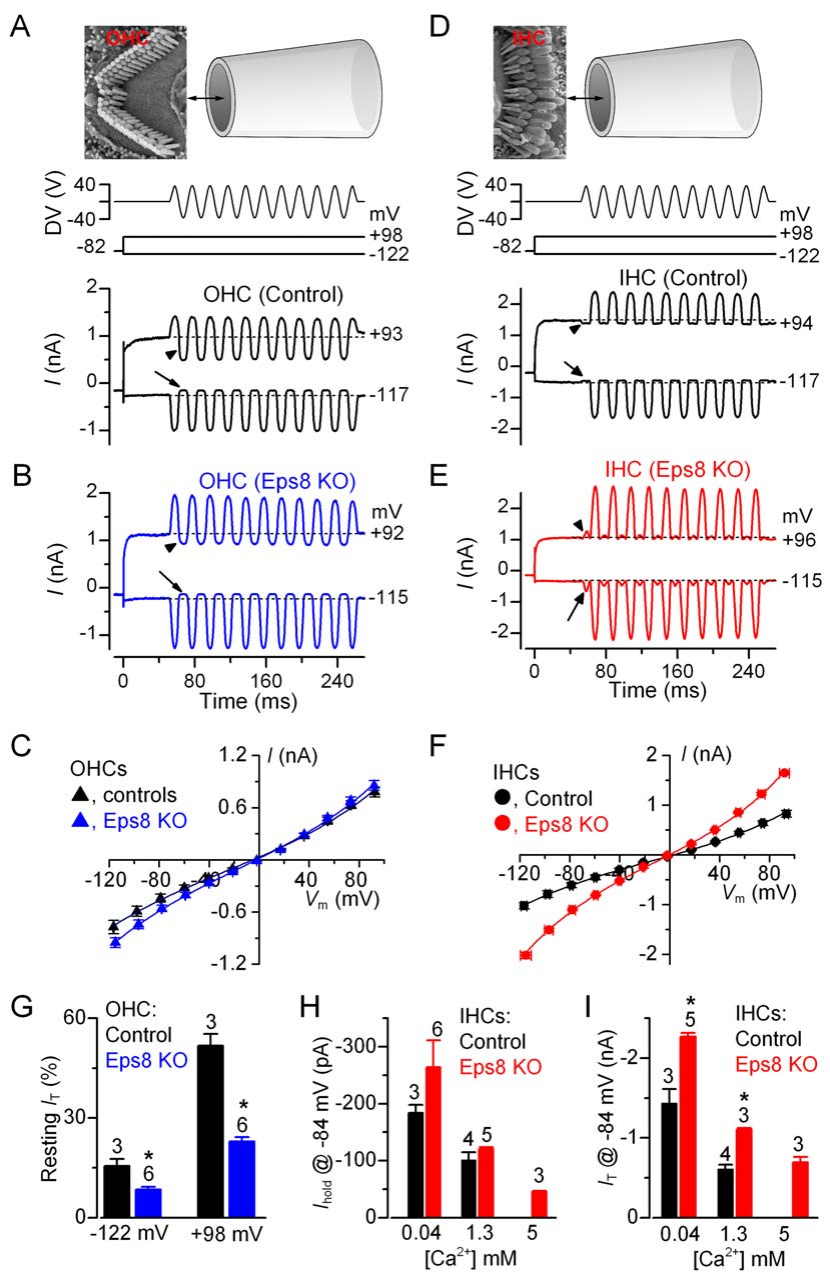
Mechano-electrical transducer current in Eps8 cochlear hair cells. (A and B) Saturating transducer currents recorded from a control (A) and a knockout (B) apical-coil Eps8 OHC by applying sinusoidal force stimuli of 50 Hz to the hair bundles. The driver voltage (DV) signal of ±40 V to the fluid jet is shown above the traces (for OHCs, negative deflections of the DV are inhibitory). The top panels show the orientation of the fluid jet (not to scale) with respect to the OHC (A) and IHC (D) hair bundle. OHC membrane potentials were stepped between −122 mV and +98 mV in 20 mV nominal increments from the holding potential of −82 mV. For clarity only a few responses are shown (membrane potentials next to the traces have been corrected by the voltage drop across the residual series resistance). The arrows and arrowheads indicate the closure of the transducer channels open at rest (i.e. resting current) elicited during inhibitory bundle displacements at hyperpolarized and depolarized membrane potentials, respectively. Note that the resting current increases with membrane depolarization. Dashed lines indicate the holding current, which is the current at the holding membrane potential. (C) Peak-to-peak current-voltage curves were obtained from three control and six knockout OHCs (P8–P9) using 1.3 mM extracellular Ca^2+^. The fits through the data are according to a simple single-energy-barrier model: *I*(*V*) = *k* [exp ((1−γ)(*V*−*V*_r_)/*V*_s_ )−exp (−γ(*V*−*V*_r_ )/*V*_s_)], where *k* is a proportionality constant, *V*_r_ is the reversal potential, *V*_s_ is a measure for the steepness of the rectification, and γ is the fractional distance within the membrane's electrical field of an energy barrier, as measured from the outside. Average parameters were obtained from fits to individual cells and were: control *k* = 284±37, *V*_r_ = −5.4±0.7 mV, *V*_s_ = 46±2 mV, and γ = 0.45±0.01; Eps8 KO *k* = 306±33, *V*_r_ = −2.4±0.3 mV, *V*_s_ = 42±2 mV, and γ = 0.45±0.01. (D and E) Saturating transducer currents recorded from a control and a knockout IHC. Note that because of the different orientation of the hair bundles in respect to the fluid jet used between OHC and IHC recordings (see top panels in A and D), negative pressure at the tip of the jet caused excitatory responses in IHCs. In knockout IHCs, a small current in the excitatory direction was elicited during inhibitory bundle displacements (this current was not present in control IHCs: arrows and arrowheads). (F) Peak-to-peak current-voltage curves were obtained from four control and three knockout IHCs (P6–P8). Control: *k* = 386±102, *V*_r_ = +1.2±1.8 mV, *V*_s_ = 51±10 mV, and γ = 0.47±0.01. EPS8 KO: *k* = 459±20, *V*_r_ = −1.3±1.3 mV, *V*_s_ = 46±1 mV, and γ = 0.48±0.01. (G) Changes in the resting transducer current at two nominal membrane potentials in control and knockout immature (P8–P9) OHCs. The resting current is given by the holding current minus the current present during inhibitory bundle deflection. (H and I) Holding current (H) and transducer current (I) at the membrane potential of −84 mV in control and knockout IHCs in the presence of 0.04 mM (endolymph-like), 1.3 mM, and 5 mM extracellular Ca^2+^. Note that 5 mM Ca^2+^ was only tested in knockout IHCs. Transducer current recordings were made at room temperature.

Mechanoelectrical transducer currents were recorded from hair cells, the hair bundles of which were stimulated with a piezo-driven fluid-jet. Transducer currents from apical-coil hair cells (P6–P9) in Eps8 control and knockout mice were elicited by alternating inhibitory and excitatory bundle displacements using 50 Hz sinusoidal force stimuli [23]. When large/saturating excitatory stimuli were applied to the bundle, a large transducer current could be recorded in all control and knockout OHCs (Figure 5A–C) and IHCs (Figure 5D–F), suggesting that the tip links observed with SEM (Figure 2) were functional. It is worth noting that the hair bundles of OHCs and IHCs were stimulated from different sides (OHCs: Figure 5A, top panel; IHCs: Figure 5D, top panel). This different approach, which was due to technical limitations of simultaneously positioning the recording patch electrode and the fluid jet with respect to the tissue, caused negative pressure at the tip of the jet to produce inhibitory responses in OHCs (Figure 5A,B) while being excitatory in IHCs (Figure 5D,E). Upon moving the bundles in the excitatory direction and at negative membrane potentials, an inward transducer current could be elicited. Any resting current flowing through open transducer channels in the absence of mechanical stimulation was reduced when bundles were moved in the inhibitory direction (i.e. away from the taller stereocilia), as was evident for control hair cells (Figure 5A and D, arrows at the negative voltage step) and knockout OHCs (Figure 5B). In contrast, in most of the knockout IHCs large inhibitory bundle displacements elicited small inward currents (Figure 5E: arrow) instead of reducing the current available at rest (Figure 5D). This anomaly was probably due to the disorganized hair bundles, where some stereocilia were oriented on the opposite side of the hair bundle compared to control cells (Figure 2B: arrows). A similar behavior has also been described in myosin VIIa mutant mice [24]. OHCs, which appeared to have a less severe bundle disorganization (Figure 2E), did not show abnormal directional sensitivity (Figure 5B). By stepping the membrane potential from −122 mV to more depolarized values, the transducer current decreased in size at first and then reversed near 0 mV (OHCs: controls, −5.4±0.7 mV, *n* = 3; knockouts −2.4±0.3 mV, *n* = 6; Figure 5C; IHCs: controls +1.2±1.8 mV, *n* = 4; knockouts −1.3±1.3 mV, *n* = 3 Figure 5F), in agreement with the non-selective permeability of MET channels to cations [25]. Note that the current became outward when excitatory bundle stimulation was applied during voltage steps positive to the reversal potential of the transducer current. At positive potentials, the larger resting transducer current, which was much more pronounced in OHCs (Figure 5A: +93 mV, arrowhead) than IHCs (Figure 5D: +94 mV, arrowhead), is likely to be due to an increased open probability of the transducer channel resulting from a reduced driving force for Ca^2+^ influx [26]. The relationship between transducer current and membrane potential shows that the maximal size of the transducer current, measured in 1.3 mM extracellular Ca^2+^, was on average 91% larger at all membrane potentials in knockout IHCs compared to that of control cells (two-way ANOVA: *p*<0.0001, Figure 5F). In OHCs the maximal size of the transducer current in Eps8 knockout mice was slightly but significantly larger than that of control cells (overall about 18% larger, *p*<0.05, Figure 5C).

We have taken advantage of the less severe hair bundle disorganization in knockout OHCs to investigate whether the absence of Eps8 had any effect on the resting transducer current. In both control and Eps8 knockout OHCs, the resting current increased with membrane depolarization as previously shown in hair cells from wild-type CD-1 mice [23]. Although the resting transducer current was significantly different between control and knockout OHCs (Figure 5G, −122 mV: *p*<0.01; +98 mV: *p*<0.0001), its increase with depolarization was the same for both genotypes (about 3 times), suggesting a similar Ca^2+^ sensitivity of the transducer apparatus. We tested whether this was also the case for IHCs by locally superfusing their hair bundle with a solution containing an endolymph-like concentration of Ca^2+^ (0.04 mM [27]). Lowering the extracellular Ca^2+^ concentration is known to increase both the maximum transducer current and its fraction activated at rest. Calcium is a permeant blocker of the transducer channel [28],[29], so the increased current amplitude in low Ca^2+^ is caused by the partial relief of this block. Moreover, extracellular Ca^2+^ causes adaptation and as such closes some transducer channels. Therefore, reducing Ca^2+^ influx into the transducer channel, by either depolarizing hair cells to near the Ca^2+^ equilibrium potential (as shown in Figure 5A,D) or lowering the extracellular concentration, causes an increased open probability of the channel [30]. In Eps8 mice, both phenomena were observed since decreasing the Ca^2+^ concentration from 1.3 mM to 0.04 mM increased the holding and maximal transducer current in both control and knockout IHCs (Figure 5H and I). Increasing the extracellular Ca^2+^ from 1.3 mM to 5 mM had the opposite effect (Figure 5H,I: tested in knockouts only). The overall change in the holding current and maximal transducer current in response to extracellular Ca^2+^ was significant in both control (*t* test: *p*<0.01 and *p*<0.005, respectively) and knockout (one-way ANOVA: *p*<0.005 and *p*<0.0001) IHCs. The maximal current, but not the holding current, was significantly larger (*P*<0.0005) in knockout compared to control IHCs when the same Ca^2+^ concentration was used (Figure 5I). The above results indicate that the biophysical properties of the transducer channel, including adaptation and the presence of a resting current, are not affected by Eps8.

The above experiments were performed on young animals (P6–P9) since this age is the most reliable for recording accurate transduction currents from mouse hair cells [31]. We tested whether transduction was likely to be functional in adult hair cells by using the styryl dye FM1-43 (Figure S5A,B). We did the same for immature cells as a comparison (Figure S5C,D). FM1-43 is a permeant blocker of the hair cell transducer channel that has previously been used to assess the presence of the resting transducer current in hair cells [23]. The advantage of this method is that the possible presence of the resting transducer current can be determined without the need of interfering with the hair bundle, thus effectively eliminating any possible artifact resulting from the abnormal orientation of stereocilia in knockout mice. Bath application or local superfusion of FM1-43 resulted in the selective labeling of immature (P7) and adult (P15–P21) control and knockout hair cells (Figure S5).

Together the above findings indicate that the biophysical properties of the transducer channel are not affected by the absence of Eps8 and that the abnormalities observed in knockout hair cells are the consequence of the disrupted hair bundle morphology, with IHCs being more affected than OHCs.

### IHCs from Eps8 Knockout Mice Do Not Develop Adult-Type Ion Channels

**Figure 6:**
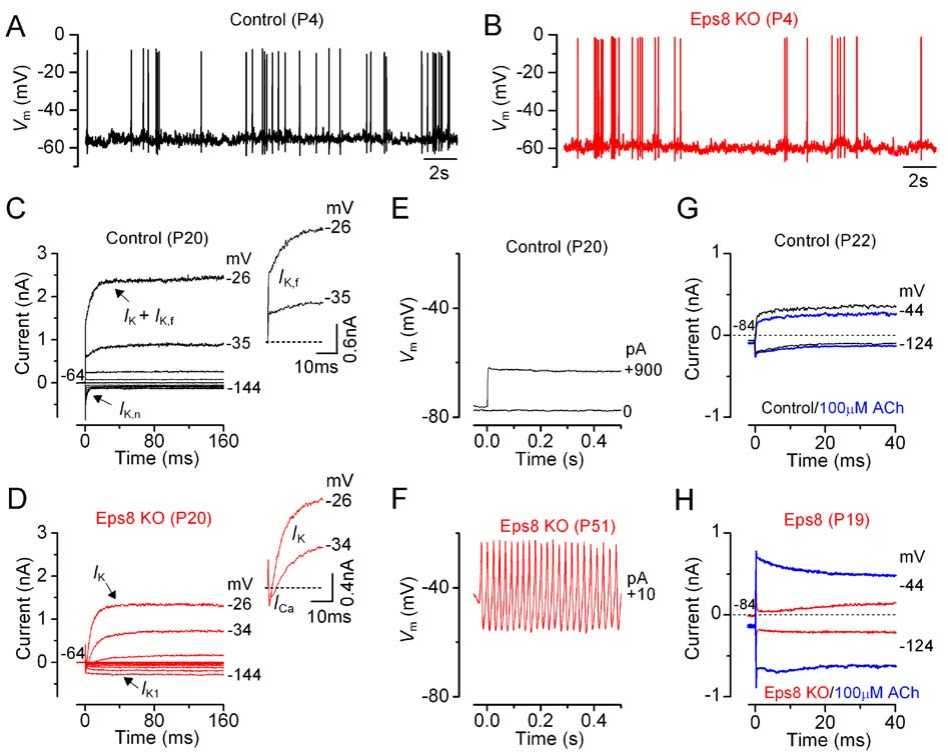
Current and voltage responses of IHCs from Eps8 mice. (A and B) Spontaneous Ca^2+^-dependent action potentials recorded from a control (A: black line) and an Eps8 knockout (B: red line) pre-hearing P4 IHC. (C and D) Currents from a control and a knockout adult P20 IHC, respectively, were elicited by depolarizing voltage steps in 10 mV nominal increments from the holding potential of −64 mV to the various test potentials shown by some of the traces. The insets show the onset (first 25 ms) of the same current recordings on an expanded scale, showing the presence of the rapidly activating *I*_K,f_ only in control cells. Note that a large Ca^2+^ current (*I*_Ca_) preceded the activation of the much slower K^+^ current (*I*_K_) in knockout IHCs. (E and F) Voltage responses induced by applying depolarizing current injections to a control and a knockout adult IHC, respectively. Note that action potentials could only be elicited in knockout IHCs. (G and H) Membrane currents recorded from adult control and knockout IHCs, respectively, before and during superfusion with 100 µM ACh (blue traces).

The biophysical properties of IHCs from Eps8 knockout mice were investigated to determine whether the loss of auditory function was associated with abnormalities in the normal function or development of these cells. In pre-hearing animals (<P12 in most rodents), the resting membrane potentials and size of K^+^ currents recorded from knockout IHCs was similar to that of controls (Table 1). Moreover, all immature IHCs investigated were able to generate spontaneous or evoked repetitive Ca^2+^ action potentials (Figure 6A,B for control and knockout IHCs, respectively) as previously shown in wild-type cells [32]. These results show that the absence of Eps8 did not interfere with the biophysical properties of pre-hearing cells.

**Table 1:** Properties of immature and adult IHCs from Eps8 knockout mice.

IHC Properties	Immature		Adult	
	P3–P8 Controls	P4–P7 Knockouts	P19–P22 Controls	P19–P51 Knockouts
Membrane capacitance (pF)	7.5±0.3 (19)	7.8±0.4 (18)	9.9±0.1 (27)	6.1±0.2 (21)
Resting potential (mV)	−57.3±1.1 (11)	−55.0±1.5 (7)	−70.9±1.6 (10)	−59.5±2.9 (11)
*I*_K1_ at −124 mV (pA)	−134±27 (7)	−139±19 (5)	—	−100±12 (10)
*I*_K_ at 0 mV (nA)	3.4±0.6 (8)	4.0±0.3 (5)	10.5±0.9 (12)	5.2±0.8 (11)
*I*_K,f_ at −25 mV (nA)	—	—	1.5±0.2 (15)	—
*I*_K,n_ at −124 mV (pA)	—	—	224±18 (6)	—

Values are means ± s.e.m.; number of hair cells is in parentheses. *I*_K1_, inward rectifier K^+^ current [35]; *I*_K_, delayed rectifier K^+^ current [34]; *I*_K,n_, negatively activated K^+^ current carried by KCNQ4 channels [34]; *I*_K,f_, Ca^2+^-activated K^+^ current [33]. “—”, not present.

We then investigated whether IHCs were able to acquire the electrical properties characteristic of adult cells, including a rapidly activating large conductance Ca^2+^-activated K^+^ current (*I*_K,f_ [33]) and a current carried by KCNQ4 channels (*I*_K,n_) with an unusually hyperpolarized activation range [34]. While both K^+^ currents were present in adult control IHCs (Figure 6C), they were absent in Eps8 knockout cells (Figure 6D; see also Table 1), which instead retained an immature phenotype by expressing the inward rectifier K^+^ current *I*_K1_ [35]. The physiological consequence of failing to acquire these adult-type currents was that Eps8 knockout IHCs retained the ability of generating slow Ca^2+^ action potentials (Figure 6F), similar to those recorded from embryonic and early postnatal IHCs [34]. They did not acquire the fast, small, and graded voltage responses (Figure 6E) as previously described in adult IHCs of normal CD-1 mice [33]. In knockout adult IHCs, the resting membrane potential (*V*_m_) was significantly more depolarized and the cell membrane capacitance (*C*_m_) significantly smaller than in control cells (Table 1: *p*<0.005 for both measurements), and similar to those found in immature cells, further supporting the role of Eps8 in IHC maturation.

We investigated the effect of the efferent neurotransmitter acetylcholine (ACh) on control and Eps8 knockout IHCs. The ACh-activated current, which is mediated by Ca^2+^ entering hair cells through α9α10-nAChRs and activating SK2 channels, is normally expressed in immature IHCs [36],[37] or adult OHCs [38] but not in adult IHCs [36],[37]. In agreement with the above findings, adult control IHCs did not respond to the extracellular application of 100 µM ACh (Figure 6G). In contrast, all knockout adult IHCs showed a large ACh-activated current at around the holding potential of −84 mV (Figure 6H), which further supports the requirement of Eps8 for their full physiological maturation. It is possible that Eps8 knockout IHCs could, to some extent, retain direct axosomatic olivocochlear efferent fibres of the auditory nerve that transiently modulate the electrical activity of pre-hearing IHCs before taking up their final position on adult OHCs [39]. Overall, the biophysical properties of the IHC membrane in Eps8 knockout mice suggest that Eps8 is required for IHC physiological maturation.

### OHC Basolateral Membrane Properties Develop Normally in Eps8 Knockout Mice

**Figure 7:**
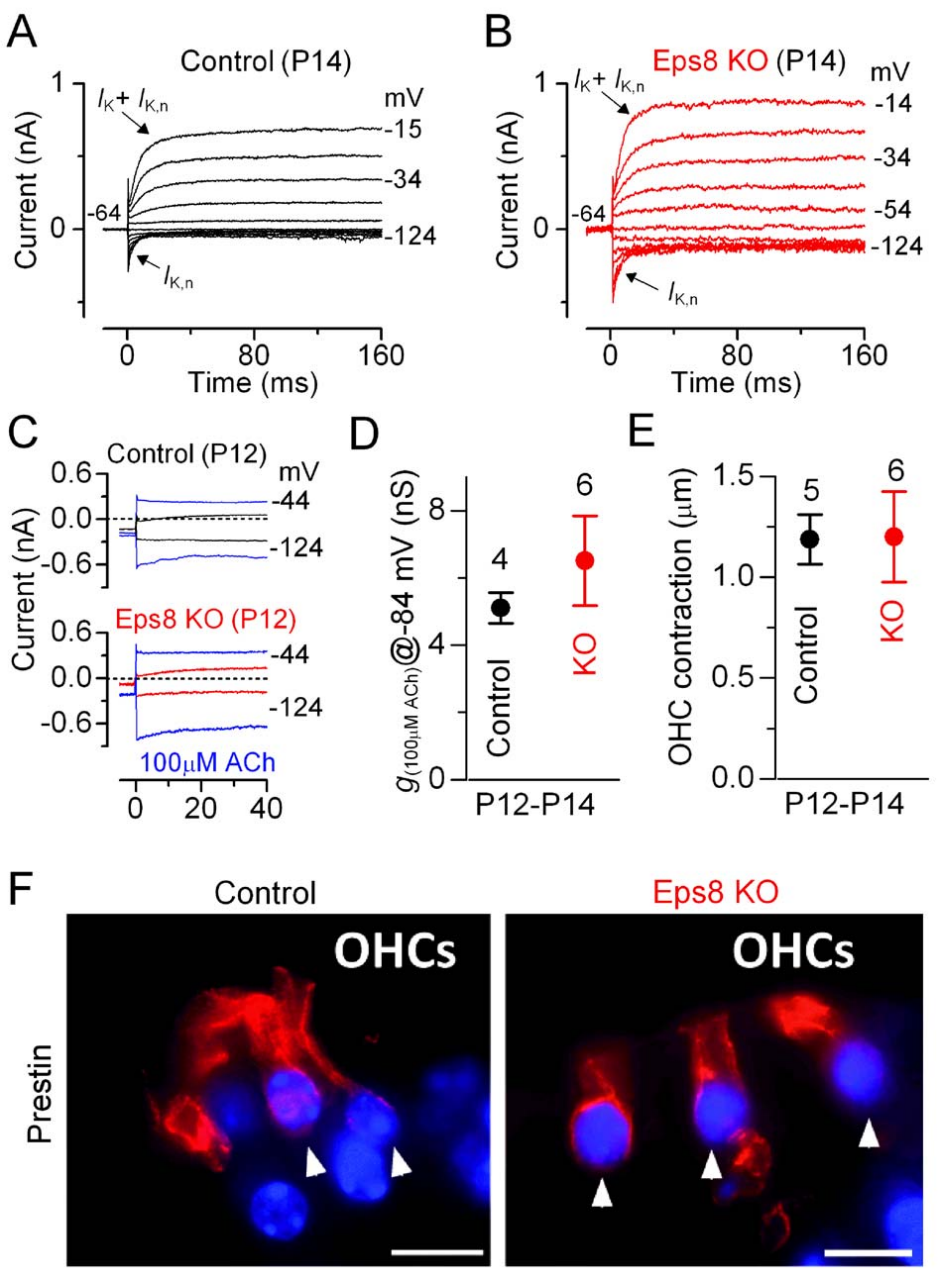
Eps8 does not affect the development of OHC basolateral properties. (A and B) K^+^ currents recorded from mature control and knockout OHCs, respectively, elicited by depolarizing voltage steps (10 mV nominal increments) from −124 mV to more depolarized values from the holding potential of −64 mV. The K^+^ current characteristic of adult OHCs, *I*_K,n_, was similarly expressed in OHCs from control and knockout Eps8 mice (see also Table 2). (C) Membrane currents recorded from control (top panel) and knockout (bottom panel) P12 OHCs before and during superfusion with 100 µM ACh (blue traces). (D) Steady-state slope conductance measured near −84 mV in the presence of 100 µM ACh. (E) OHC contraction (i.e. electromotility) in response to voltage steps from −64 to +56 mV at room temperature. Note that the size of the total K^+^ current, the isolated *I*_K,n_ (see also Table 2), ACh responses, and electromotile activity were all similar between control and knockout OHCs. (F) Immunolabeling of the motor protein prestin (red) in OHCs of adult control (left panel) and Eps8 knockout mice (right panel) was normal. Arrowheads point to OHCs. Scale bars: 10 µm.

We investigated immature and adult Eps8 knockout OHCs to determine whether the development of their basolateral membrane properties was affected as in IHCs. Immature OHCs from knockout mice exhibited biophysical characteristics similar to those measured in control cells (Table 2). However, in contrast to IHCs, the resting membrane potential and cell membrane capacitance of adult OHCs were found to be similar between control and knockout cells (Table 2). *I*_K,n_, the major current component in adult mouse OHCs and normally expressed from P8 onwards [40], was seen in all adult OHCs investigated (Figure 7A,B; Table 2), and its size, measured in isolation as previously described [40], did not differ significantly between control and knockout cells (Table 2). Moreover, adult knockout OHCs were sensitive to ACh (Figure 7C,D), exhibited electromotile activity (Figure 7E), and expressed the motor protein prestin [41] in their basolateral membrane (Figure 7F). Despite the fact that *I*_K,n_ and ACh-mediated responses in IHCs and OHCs are carried by the same channels, their normal developmental expression was only affected in IHCs. These findings indicate that although IHCs and OHCs share some similar biophysical properties, only the maturation of IHCs appeared to be affected by the absence of Eps8.

**Table 2:** Properties of immature and adult OHCs from Eps8 knockout mice.

OHC Properties	Immature		Adult	
	P3–P9 Controls	P5–P8 Knockouts	P10–P14 Controls	P10–P14 Knockouts
Membrane capacitance (pF)	6.4±0.3 (13)	6.2±0.4 (10)	6.5±0.3 (15)	7.1±0.4 (17)
Resting potential (mV)	−52.5±2.1 (3)	(0)	−56.7±1.9 (6)	−56.4±1.9 (5)
*I*_K_ at 0 mV (nA)	1.8±0.1 (10)	1.9±0.2 (3)	1.2±0.1 (7)	1.5±0.1 (17)
*I*_K,n_ at −124 mV (pA)	—	—	109±10 (4)	164±21 (16)

Values are means ± s.e.m.; number of hair cells is in parentheses. *I*_K_, delayed rectifier K^+^ current [40]; *I*_K,n_, negatively activated K^+^ current carried by KCNQ4 channels [40].

### Exocytosis in IHCs from Eps8 Knockout Mice Fails to Mature

**Figure 8:**
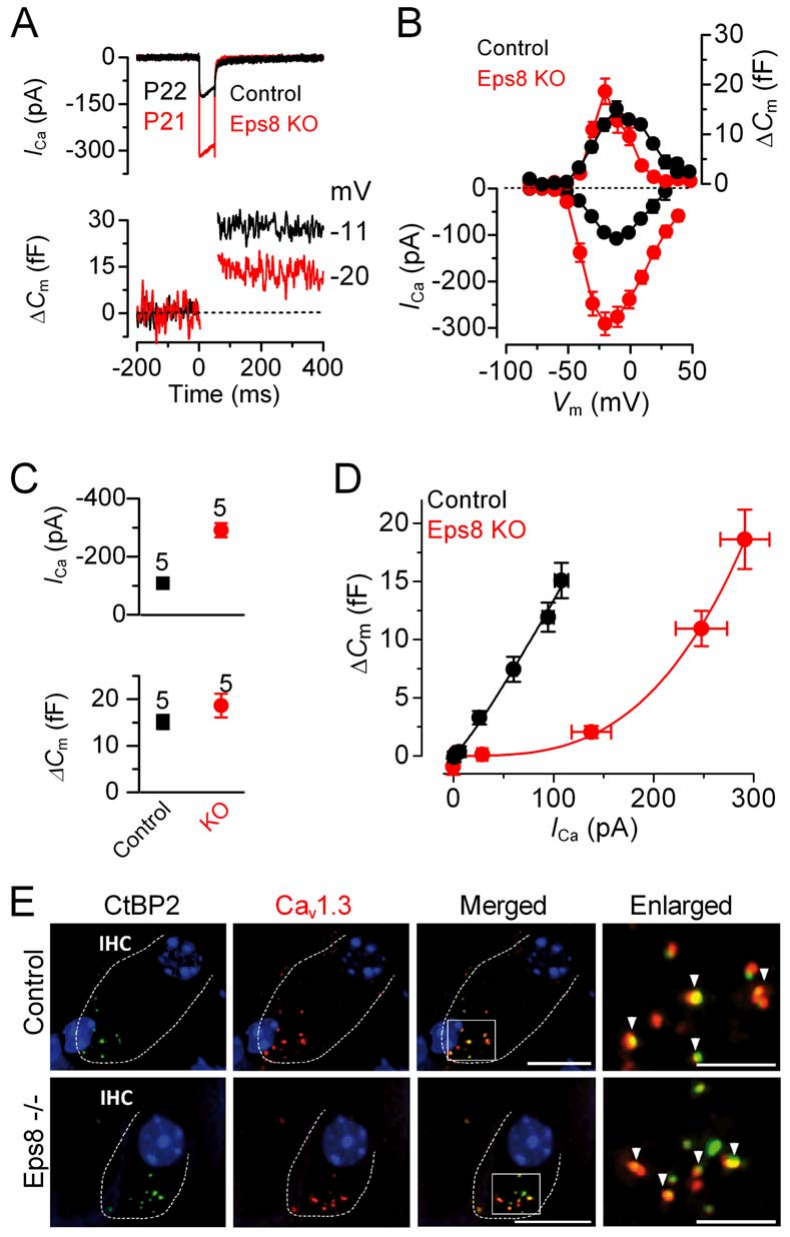
Exocytotic Ca^2+^ dependence and synaptic organization in Eps8 IHCs. (A and B) *I*_Ca_ and ΔC_m_ responses from adult control and Eps8 knockout IHCs. Recordings were obtained in response to 50 ms voltage steps, in 10 mV increments, from −81 mV. For clarity, only maximal responses are shown in (A). (C) Maximal peak *I*_Ca_ (top panel) and Δ*C*_m_ (bottom panel) values, from P22 control and P21 knockout IHCs. (D) Synaptic transfer relations obtained by plotting Δ*C*_m_ against the corresponding *I*_Ca_ between −71 mV and the peak *I*_Ca_ from panel B, showing that knockout IHCs exhibited a steeper intrinsic Ca^2+^ dependence of exocytosis than control cells [42]–[45]. Fits are according to eqn. 1 (see Methods). (E) Immunolabeling of synaptic ribbons (green: CtBP2/RIBEYE) and Ca_V_1.3 Ca^2+^ channels (red) at the presynaptic site of control (top panels) and knockout (bottom panels) adult IHCs (enlarged images magnified in the right panels). A comparable degree of colocalization was present in IHCs from both genotypes (indicated by the yellow overlapping staining and arrowheads in the right panels). Scale bars: 10 µm, apart from the two enlarged panels, where they are 5 µm.

Since the K^+^ currents did not mature in knockout IHCs, we investigated whether the development of *I*_Ca_ and the induced exocytosis were altered in the absence of Eps8. Exocytosis was estimated by measuring increases in cell membrane capacitance (Δ*C*_m_) following depolarizing voltage steps, which is generally interpreted as an indication of neurotransmitter release from presynaptic cells. The synaptic machinery of IHCs becomes more sensitive to Ca^2+^ as they mature, causing synaptic vesicles to be released linearly with increases in Ca^2+^ current [42]–[45]. We found that in the absence of Eps8 the developmental linearization of the exocytotic Ca^2+^ sensitivity in IHCs did not occur. In adult Eps8 knockout IHCs the maximal size of the Ca^2+^ current (*I*_Ca_) was significantly larger (*p*<0.0001) than that of control cells (Figure 8A–C) but similar to that measured in pre-hearing cells [42]–[45]. However, the corresponding Δ*C*_m_ was similar between the two genotypes (Figure 8B,C, lower panel). As a consequence the exocytotic Ca^2+^ dependence, defined as the variation in Δ*C*_m_ as a function of *I*_Ca_ and displayed as a synaptic transfer function [42]–[45], was significantly less linear in the knockout (power of 3.4±0.6, *n* = 5) than in control (power of 1.2±0.1, *n* = 5: Figure 8D) adult IHCs and was instead comparable to that of immature cells [42],[44]. Despite these physiological abnormalities, adult knockout IHCs appeared to show a normal distribution of both Ca^2+^ channels and synaptic ribbons (Figure 8E). In order to account for the larger Ca^2+^ current in knockout IHCs, it is likely that either the Ca^2+^ channel density per spot is larger or single Ca^2+^ channel properties, such as open probability and regulation, have been affected.

## Discussion

We have shown that Eps8 is located in the tips of the stereocilia in mammalian IHCs and OHCs and that it is required for regulating hair bundle length, which is crucial for the normal mechano-sensitivity of the transduction apparatus. We have also shown that in the absence of Eps8 IHCs remain immature at the onset of hearing (Figure S6). Interestingly, Eps8 appeared not to influence OHC maturation, despite the fact that they have almost identical developmental requirements as IHCs, including the expression of similar K^+^ channels and responses to the efferent neurotransmitter ACh. The relevance of this molecule to hearing is emphasized by the fact that mice lacking Eps8 are profoundly deaf.

### Eps8 Is Required for the Elongation and Maintenance of Cochlear Hair Cell Stereocilia

Transduction of acoustic stimuli into electrical signals relies on hair bundles being oriented along the axis of mechano-sensitivity, which is critical for the optimal opening of transducer channels [1]. The maturation and maintenance of hair bundle height requires continuous turnover of the actin filaments that form the core of each stereocilium [4],[46]. Several stereociliary proteins are known to contribute to the normal development and/or maintenance of hair bundle structure and function [2],[47], but none of these appear to exert direct control over actin polymerization in hair cell stereocilia. Recently it has been shown that twinfilin 2, an F-actin barbed-end-capping and G-actin-sequestering protein that can inhibit actin polymerization, is expressed at the tips of the shorter stereocilia of IHCs and OHCs. Overexpression of twinfilin 2 in cultured IHCs resulted in a significant reduction of stereocilia length, suggesting that twinfilin 2 limits elongation of the shorter stereocilia in order to maintain the mature staircase architecture of cochlear hair bundles [3]. Gelsolin is another actin capping protein present at the tip of the shorter stereocilia on OHCs and is crucial for regulating their elongation [48].

Here we show that Eps8 is expressed at the tips of stereocilia in mammalian cochlear IHCs and OHCs, which is in agreement with recent findings in OHCs [49]. The absence of Eps8 results in abnormally short stereocilia, particularly those in the tallest row, with IHCs being substantially more affected than OHCs. Therefore, in contrast to twinfilin 2 and gelsolin, Eps8 appears to favor stereocilia elongation. Eps8, like twinfilin 2 and gelsolin, is an actin capping protein able to inhibit the growth of actin filaments at their plus end [17],[50], but it also acts as a cross-linking or bundling protein and regulates microvillar morphogenesis in *Caenorhabditis elegans* [18]. Actin-bundling-proteins are required for forming protrusions such as filopodia and microvilli in eukaryotes [51]. Interestingly, Eps8 can switch between capping and bundling activity, with capping activity activated by Abi-1 [50] and bundling by IRSp53 [52]. Recent evidence has indicated that the capping activity of Eps8 is required for the formation of filopodia in hippocampal neurons [19]. Although Eps8 has been shown to regulate the length of intestinal microvilli in mice [21], it is not known whether it controls this via its capping and/or bundling function. The identity of Eps8 interaction partners in hair cells is not known, although our data, together with that of a recently published study [49], are consistent with similar activities for Eps8 in hair cell stereocilia growth and maintenance.

### Eps8 Is Not Required for the Mechano-Electrical Transducer Current

Scanning electron microscopy of Eps8 knockout hair cells revealed the presence of tip links, which are thought to be a pre-requisite for mechano-electrical transduction [53]. We found that large transducer currents could be elicited following saturating hair-bundle stimulation in the absence of Eps8, suggesting that the observed tip links are functional and that this novel stereociliary protein is not essential for mechano-electrical transduction in cochlear hair cells. The larger transducer current recorded in Eps8 knockout hair cells, with IHCs being more affected than OHCs, could be explained by the presence of more functional transducer channels per hair bundle. This is conceivable considering the presence of the additional rows of stereocilia, especially in knockout IHCs. If we assume that all additional stereocilia on knockout IHCs have functional transducer channels connected by tip links, then there would be twice the number of transducer channels per bundle, which would account for the larger transducer current. The presence of the resting transducer current and the similar Ca^2+^ sensitivity of the channel in control and knockout hair cells (Figures 5 and S5) indicate that Eps8 is not involved in determining the biophysical properties of the transducer channel. This is not true for many other proteins associated with hair bundles and hearing loss [2],[54] including myosin VIIa [24], myosin XVa [55], and harmonin-b [56]. IHCs from myosin XVa mutant mice have short hair bundles similar to those in Eps8 mice [55], but they also have a series of additional defects in the transducer apparatus, including absence of tip links and loss of adaptation and Ca^2+^ sensitivity of the transducer current [55]. Nevertheless, the short hair bundles in Eps8 knockout hair cells could have a large impact in adult animals because the relationship between force applied and hair bundle displacement could compromise the ability to detect physiological sound pressures. This scenario is suggested by the CAP I/O relation (Figure S2C) and in particular by the absence of CM responses in knockout mice (Figure S4). The latter indicates that the much smaller hair bundles of OHCs (Figure 2; see also [49]), which was the only abnormality observed in these cells, are unlikely to be coupled to the tectorial membrane and therefore would not be stimulated effectively in normal physiological conditions.

### The Absence of Eps8 Prevents the Normal Maturation of Cochlear IHCs

Prior to the onset of hearing (postnatal day 12 in most rodents) immature hair cells of the mammalian cochlea follow a developmental program that includes the acquisition and/or elimination of different basolateral membrane proteins (e.g. ion channels and synaptic molecules [57]) in order to mature into fully functional sensory receptors. These proteins in adult IHCs are directly involved in shaping the receptor potential generated by the opening of the transducer channels and in triggering exocytosis at hair cell ribbon synapses [58]. Although the physiology of hair cells changes progressively through development (Figure S6), the most significant and abrupt transition occurs at the onset of hearing [57]. The prevailing hypothesis is that hair cell functional maturation is controlled by a developmental switch, which is thought to be influenced by spontaneous Ca^2+^ action potential activity [32],[34],[59].

We found that, although the basolateral properties of immature IHCs were indistinguishable between control and Eps8 knockout mice, in adult cells the absence of Eps8 was accompanied by morphological differences, such as a smaller cell size and apparent persistence of axo-somatic efferent connections. There was also a failure in the normal appearance of mature biophysical characteristics, namely K^+^ currents (*I*_K,f_, *I*_K,n_) [32],[34], linear exocytotic Ca^2+^ dependence [42]–[45], and the down-regulation of immature-type channels such as those carrying *I*_K1_, *I*_Ca_, and ACh-responses (Figure S6 [32],[35],[37]. The possibility that an absence of Eps8 only delays the normal physiological maturation of cochlear hair cells, as previously described in mice lacking thyroid hormone receptors [60], is unlikely since the observed defects persisted in IHCs from nearly 2-mo-old Eps8 knockout mice.

Calcium-dependent action potential activity in immature IHCs is required for the expression of the BK current *I*_K,f_ [61] and the linear exocytotic Ca^2+^ dependence [62] in adult cells. It is also possible that IHC maturation depends on the presence of the resting transducer current in immature cells, especially from the second postnatal week when the endocochlear potential begins to increase [63], since it would depolarize the hair cells and affect the action potential activity. However, the biophysical properties of pre-hearing Eps8 knockout IHCs, including action potential activity and resting mechano-electrical transducer current, appeared similar to those of control cells. This excludes the possibility that in Eps8 knockout mice the developmental switch between immature and adult IHCs is prevented by a functional defect in IHCs during immature stages. Despite the similarities between hair cells, the fact that OHCs appear unaffected in the absence of Eps8 suggests that their functional maturation is regulated somewhat differently to that of IHCs.

The unconventional myosin VI, which when mutated causes hereditary deafness in mice (*Snell's waltzer*) and humans [54],[64], is a protein responsible for actin-based motility. In hair cells myosin VI is required for the normal developmental expression of adult-like ion channels/presynaptic proteins, most likely by affecting intracellular trafficking [65],[66]. However, in contrast to Eps8 knockout mice, the hair-cell hair bundles in *Snell's waltzer* mice are profoundly disorganized [67], which is likely to affect the biophysics of mechanoelectrical transduction (i.e. resting transducer current and adaptation) as also previously shown in myosin VIIa mutants [24]. A similar phenotype to that of Eps8 knockout mice has been observed in mutant mice lacking the transmembrane protein *Tmc1* [68], but in this case both IHCs and OHCs failed to mature. *Tmc1* has been suggested to be involved in intracellular trafficking or, more generally, in the activation or modulation of intracellular signals associated with hair cell maturation [68]. Based on current experimental evidence, a similar effect on hair cell maturation to that of *Tmc1* could also be postulated for Eps8, whether localized in the stereocilia or in the cytoplasm (Figure 1). Currently, little is known about the specific role of Eps8 in actin remodeling in mammals. In mice, it has been shown that the absence of Eps8 causes the cytoskeleton to be more refractory to actin depolymerization (i.e. a less dynamic cytoskeleton) and a larger NMDA current in cerebellar granule cells [20]. Eps8 activity has also been described to regulate actin dynamics in response to extracellular (e.g. BDNF [19]) and intracellular (e.g. IRSp53 [52]) factors, suggesting that the absence of IHC maturation in knockout mice could originate from a more general/indirect role of Eps8 in regulating developmental signaling at around the onset of hearing.

Mutant mice have proven to be a powerful means for identifying the molecular mechanisms responsible for the development and maintenance of normal hearing [2],[47],[64]. We found that in the absence of Eps8 mice are deaf and that, in the cochlea, the protein directly influences stereocilia growth and is required for IHC maturation. The exact mechanism by which Eps8 is able to control such a complex developmental program remains a major challenge for future studies.

## Materials and Methods

### Single-Hair Cell Electrophysiology

Inner and outer hair cells (IHCs: *n* = 82; OHCs, *n* = 58) from Eps8 mutant mice [20] were studied in acutely dissected organs of Corti from postnatal day 3 (P3) to P51, where the day of birth is P0. Animals were killed by cervical dislocation in accordance with UK Home Office regulations. Cochleae were dissected as previously described [42]–[45] in normal extracellular solution (in mM): 135 NaCl, 5.8 KCl, 1.3 CaCl_2_, 0.9 MgCl_2_, 0.7 NaH_2_PO_4_, 5.6 D-glucose, 10 Hepes-NaOH, 2 sodium pyruvate, amino acids, and vitamins (pH 7.5; osmolality ∼308 mmol kg^−1^). Superfusion of hair cells with 100 µM ACh (Sigma) was performed with a multi-barreled pipette positioned close to the patched cells. Unless otherwise stated, all recordings were performed near body temperature (35–37°C) and using 1.3 mM Ca^2+^ in the extracellular solution. All animals were genotyped as previously described [20].

Voltage and current recordings were obtained using the following intracellular solution (in mM): 131 KCl, 3 MgCl_2_, 1 EGTA-KOH, 5 Na_2_ATP, 5 Hepes-KOH, 10 sodium phosphocreatine (pH 7.3). The pipette intracellular solution for exocytosis measurements contained (in mM): 106 Cs-glutamate, 20 CsCl, 3 MgCl_2_, 1 EGTA-CsOH, 5 Na_2_ATP, 0.3 Na_2_GTP, 5 Hepes-CsOH, 10 Na_2_-phosphocreatine (pH 7.3); that for mechano-electrical transduction contained (in mM): 135 CsCl, 2.5 MgCl_2_, 1 EGTA-CsOH, 2.5 Na_2_ATP, 10 sodium phosphocreatine, 5 Hepes-CsOH (pH 7.3). Patch pipettes were coated with surf wax (Mr. Zogs SexWax, USA) to minimize the fast patch pipette capacitance transient. Electrophysiological recordings were made using Optopatch (Cairn Research Ltd, UK) or Axopatch 200B (Molecular Devices, USA) amplifiers. Data acquisition was controlled by pClamp software using Digidata 1320A or 1440A boards (Axon Instruments, CA, USA). Recordings were low-pass filtered at 2.5 kHz (8-pole Bessel) or 2.0 kHz (4-pole Bessel), sampled at 5 kHz, and stored on computer for off-line analysis (Origin: OriginLab, USA). Membrane potentials were corrected for the *R*_s_ (IHCs: 3.5±0.3 MΩ, *n* = 74; OHCs: 3.4±0.3 MΩ, *n* = 54) and liquid junction potential (Cl- and Glutamate-based intracellular solution: −4 mV and −11 mV, respectively). The overall average voltage-clamp time constant (product of *R*_s_ and membrane capacitance *C*_m_) was 27±3 µs for IHCs (*n* = 74) and 24±2 µs for OHCs (*n* = 54).

Real-time changes in membrane capacitance (Δ*C*_m_) were measured using the Optopatch as previously described [42]–[45]. Briefly, a 4 kHz sine wave of 13 mV RMS was applied to IHCs from −81 mV and was interrupted for the duration of the voltage step. The capacitance signal from the Optopatch was amplified (×50), filtered at 250 Hz, and sampled at 5 kHz. Capacitance changes were measured by averaging the *C*_m_ traces after the voltage step (around 200 ms) and subtracting the pre-pulse baseline. Δ*C*_m_ was recorded while applying 30 mM TEA and 15 mM 4AP (Fluka, UK) and 60 µM linopirdine to reduce K^+^ currents. The relation between Ca^2+^ entry and exocytosis in IHCs (Figure 8D), estimated using a synaptic transfer function [42]–[45], was obtained by plotting Δ*C*_m_ against the peak *I*_Ca_ for 50 ms voltage steps from −71 mV to that where the maximal *I*_Ca_ occurred from the *I*-V curves (Figure 8B). Data were approximated using a power function: 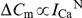 (eqn. 1), where N is the power.

Mechano-electrical transduction was recorded by mechanically stimulating the hair bundles of immature apical-coil IHCs using a solution-filled pipette (8–10 µm tip diameter) inserted into a piezoelectric disc-driven fluid jet as described before [23]. The pipette tip was positioned near to the bundles to elicit a maximal transducer current. Saturating mechanical stimuli were applied as 50 Hz sinusoids (filtered at 0.25 kHz, 8-pole Bessel) with ±40 V driving voltages. For these experiments we used KCl- or CsGlutamate-based intracellular solution. Voltage clamp protocols are referred to a holding potential of −84 mV or −82 mV. The effect of endolymph-like Ca^2+^ (20–40 µM: [27]) was examined by using a solution containing low Ca^2+^ (40 µM Ca^2+^, buffered with 4 mM HEDTA). During the experiments in which different extracellular Ca^2+^ concentrations were used (0.04 mM, 1.3 mM, or 5 mM), the hair bundles were topically superfused and the fluid jet pipette was also filled with the same solution.

The presence of electromotile activity in OHCs was estimated by applying a depolarizing voltage step from the holding potential of −64 mV to +56 mV and cell length change recorded using a CCD camera, with a ×3 magnifier, attached to the microscope (×63 objective). The aim of this experiment was to assess whether Eps8 knockout OHCs retained their electromotile ability. OHC contraction was visually obvious and was typically measured as length change between the patch electrode (positioned just below the cuticular plate) and the nucleus region. Measurements were performed in Photoshop and were calibrated using a microscope grid (20 µm = 520 pixels). Our data (control: 1.19±0.12 µm, *n* = 5; knockout: 1.20±0.22 µm, *n* = 6) are in agreement with previous recordings using a similar technique (13 nm/mV [69], giving 1.56 µm contraction for a similar 120 mV range to that used in our study).

Statistical comparisons of means were made by Student's two-tailed *t* test or, for multiple comparisons, analysis of variance, usually one-way ANOVA followed by the Tukey test. Mean values are quoted ± s.e.m. where *p*<0.05 indicates statistical significance.

### FM1-43 Labeling

Stock solutions of 1 or 3 mM FM1-43 (n-(3-triethylammoniumpropyl)-4-(4-(dibutylamino)styryl) pyridiniumdibromide: Molecular Probes or Invitrogen) were prepared in water. FM1-43 dye labeling was studied using bath or topical application methods. After dissections the apical coils of control and knockout cochleae (aged P7–P21) were immobilized at the bottom of a microscope chamber containing normal extracellular solution. Cochleae were then bathed or superfused with a solution containing 3 µM FM1-43 for 10–15 s and immediately washed several times with normal extracellular solution. The cochleae were then viewed with an upright microscope equipped with epifluorescence optics and FITC filters (excitation 488 nm, emission 520 nm) using a 63× water immersion objective. Images were captured within 10–20 min from the dye application using a CCD camera (SPOT-JNR). Some experiments were also performed using a confocal microscope. A total number of 20 control and 15 knockout cochleae from 11 and 8 mice, respectively, were used. These experiments were performed at room temperature (22–25°C) as previously described [23].

### In Vivo Hearing Tests and Electrocochleography

All in vivo measurements were performed on anesthetized adult mice (7 controls and 8 knockouts).

*Auditory brainstem responses* (ABRs) were performed as previously described [70]. Briefly, to record auditory brainstem responses, subdermal silver-wire electrodes were inserted at the vertex (reference) and ventro-lateral to the measured ear (active) and the back of the animal (ground). Responses were measured for click and noise burst stimuli and stimulus frequencies between 2.0 and 45.2 kHz in 2 steps per octave. Responses were amplified by 94 dB and band pass filtered between 0.2 and 5 kHz. Stimulus sound pressure levels were typically 20–100 dB SPL, presented in steps of 5 dB.

#### Distortion product otoacoustic emissions (DPOAE)

Outer hair cell function was assessed using DPOAE as previously described [70].

*In vivo electrocochleography* (compound action potentials of the auditory nerve, CAP; summating potential of IHC receptor potentials, SP; cochlear microphonic, CM) was performed in anesthetized mice. In addition to the general anesthesia, lidocaine hydrochloride (Xylocain 2%, AstraZeneca, Wedel, Germany) was subcutaneously injected at sites of surgical intervention. The bony bulla was exposed retro-auricularly through a surgical cut in the skin and a 1 mm hole drilled through the bone at the top end of the bulla, close to the ear canal. The mucosa within the hole was removed and the round window niche visualized, just above the stapedial artery. A silver ball recording electrode was placed on the round window membrane. Subdermal silver wire electrodes were inserted at the vertex (reference) and the back (ground) of the animal. Sound-evoked electrical potentials (i.e. CAP, CM, and SP) were recorded by the active electrode positioned on the round window. CAPs were recorded in free field with click stimuli (100 µs, alternating condensation and rarefaction) and frequency-specific tone bursts (3 ms, 1 ms ramp, alternating phase, 2 kHz to 45 kHz in 1–4 steps per octave, sound pressure levels were below threshold up to 120 dB SPL in 5 dB steps). The electrode signals were averaged for 8–32 repetitions at a stimulus repetition rate of 40/s. The signal was amplified (74 dB), filtered (0.2–5 kHz), and added for alternating phase or polarity to omit the stimulus artifact and cochlear microphonics. SPs were obtained with 20 ms tone bursts, responses filtered from 5 Hz to 5 kHz, averaged 512 times. CMs were recorded for 10 ms tone bursts band pass filtered from 0.2 to 50 kHz, averaged 64 times for stimuli of 4 kHz, 11.3 or 16 kHz, and 32 kHz and sound pressure levels from below threshold to 80 dB SPL in steps of 10 dB.

#### Input/output functions

From the waveform signal of the CAP and SP recordings, peak amplitudes (in µV) were calculated and plotted as I/O functions with increasing stimulus level (0–120 dB SPL). For CAP I/O function, peak-to-peak amplitudes were calculated for the stimulus frequency of 16 kHz (control mice: positive 0.8 ms, negative 1.4 ms, positive 1.9 ms; Eps8 knockout mice: negative 1.1, positive 1.4, negative 1.8). For SP, amplitude was defined as average DC potential minus baseline. SP amplitudes were calculated for stimulus frequencies giving the best response; i.e. 11.31 kHz for controls and 16 kHz for Eps8 knockout mice.

#### Endocochlear potential (EP)

In anesthetized mice the bony bulla was exposed via a ventral approach. Surgery was kept to minimum to avoid the loss of blood or reduced oxygen supply. After opening the bulla wall above the basal turn of the cochlea using a 1 mm diameter drill, the bony wall above the *stria vascularis* was thinned out until only a soft tissue layer was covering the endolymphatic space of the *scala media*. The EP was measured while penetrating into the *scala media* using a glass pipette microelectrode (tip diameter about 1 µm, filled with 150 mM KCl). EP was calculated at a depth from 120 to 420 µm (drift in the potentials was typically below 10 mV).

CAP and SP, CM, DPOAE, and ABRs were performed under anesthesia and the experimental protocols were reviewed and approved by the animal welfare commissioner and the regional board for scientific animal experiments in Tübingen (Germany). Experiments were performed in a soundproof chamber (IAC) as described [70]. For stimulus generation and signal recording, a multi-function IO-Card (PCI-6052E or PCI-MIO-16-E1, NI, USA) was used. Sound pressure level was controlled with custom made attenuators and amplifiers (Wulf Elektronik, Germany). Stimuli were delivered to the ear by calibrated loudspeakers (DT-911, Beyerdynamic, Germany) placed 3 cm lateral to the animal's pinna or in closed field (DPOAE). Sound pressure was calibrated online prior to each measurement with a microphone probe system (B&K 4191, Bruel & Kjaer, Denmark). Thresholds were defined as the sound pressure level where a stimulus-correlated response was clearly identified by visual inspection of the averaged signal.

Statistical comparisons of means were made by two-way ANOVA. Mean values are quoted ± s.d. where *p*<0.05 indicates statistical significance.

### Immunofluorescence Microscopy

Cochleae from immature (P5–P7) and adult (P45–P46) control mice (NMRI, C57BL/6, Eps8) and Eps8 knockout mice were used to prepare cryosections or whole-mount preparations for immunofluorescence microscopy and processed as previously described [65]. Briefly, cochleae prepared for cryosections were dissected and fixed for 2 h with 2% paraformaldehyde, decalcified, embedded in Tissue-Tek optimal cutting temperature compound, and cryosectioned at a thickness of 10 µm. Sections were embedded with Vectashield mounting medium with DAPI (Vector Laboratories). For whole-mount preparations, the whole organs of Corti were dissected out in 1×PBS and mounted on slides. Antibodies to Eps8 (mouse, monoclonal, BD Transduction Laboratories, diluted 1∶50), otoferlin (rabbit, diluted 1∶6000 [71]), Ca_V_1.3 (rabbit, Alomone Laboratories, diluted 1∶50), prestin (rabbit, diluted 1∶3000 [72]), and CtBP2/Ribeye (mouse, BD Transduction Laboratories, diluted 1∶50) were used. Primary antibodies were detected with Cy3-conjugated (Jackson ImmunoResearch Laboratories) or Alexa Fluor 488–conjugated secondary antibodies (Molecular Probes). Sections were viewed using an Olympus BX61 microscope equipped with motorized *z* axis, epifluorescence illumination, and differential interference contrast (DIC). Images were acquired using a CCD camera and analyzed with cellSense Dimension software (OSIS). To display Ca^2+^ channel and ribbon distribution, cochlear slices were imaged over a distance of several µm with the coverage of the IHC synaptic region in an image-stack along the *z* axis (z stack) followed by 3-dimensional deconvolution using cellSense Dimension module with the advanced maximum likelihood estimation algorithm (ADVMLE, OSIS). Figure 7F shows composite images, which represent the maximum intensity projection over all layers of the z stack. Images were processed with Photoshop. The distribution of Eps8 and prestin in apical coil hair cells was determined in at least three animals of a given age and done at least in triplicate on each. For confocal microscopy, whole-mount cochleae were counterstained for F-actin with Alexa 568 (1∶400 dilution) conjugated phalloidin (1∶1000 dilution) or labeled with the IHC marker VGLUT3 (rabbit, 1∶200 dilution: Synaptic System) [65], mounted in Vectashield, and viewed with a Zeiss 510 Meta confocal laser scanning microscope.

### Scanning Electron Microscopy

For SEM cochleae were excised from wild-type, including CD-1 mice, and knockout Eps8 mice (P2–P5 and P18) and a hole made in the apex. They were fixed by intralabyrinthine perfusion using a fine hypodermic needle through the round window with 2.5% glutaraldehyde in sodium cacodylate buffer containing 2 mM calcium chloride (pH 7.4) and then immersed in this fixative for 2 h. They were stored in fixative diluted 1/10^th^ in buffer and subsequently dissected by removing the bone from the apical coil to expose the organ of Corti and then immersed in 1% osmium tetroxide in the same cacodylate buffer for 1 h. For osmium impregnation, which avoids gold coating, cochleae were incubated in solutions of saturated aqueous thiocarbohydrazide (20 min) alternating with 1% osmium tetroxide in buffer (2 h) twice (the OTOTO technique [73]). They were dehydrated through an ethanol series and critical point dried using CO_2_ as the transitional fluid, then mounted on specimen stubs using silver conducting paint (Agar Scientific, Stansted, UK), and examined in a Hitachi S4500 field emission SEM operated at 5 kV accelerating voltage. Images were obtained from >10 control and four knockout mice.

### Transmission Electron Microscopy and Immunogold Electron Microscopy

For TEM cochleae from adult Eps8 knockout and wild type mice (P22, P26, and P35) were fixed as for SEM, the shell was partially removed from both sides of the spiral, and the cochleae postfixed by immersion for 1 h in 1% osmium tetroxide in the cacodylate buffer, dehydrated, and embedded in Spurr resin using a standard protocol [74]. The apical region was exposed by cutting longitudinally through the centre of the modiolus using an annular diamond blade on a Malvern Instruments (Malvern, UK) 2A microslicer. Semi-thin serial sections (250 nm) were cut from the apical coil and mounted on copper hole grids coated with a formvar film. They were examined unstained in a JEOL 1230 TEM operated at 100 kV accelerating voltage. To measure the height of the stereocilia, digital images were acquired using a Megaview III (SIS systems, Olympus Microscopes Ltd) and stereocilia were selected for measuring provided the tips and more than 75% of the shaft were evident in the section. The majority of sections contained the whole length of stereocilia. Measurement was performed using the “arbitrary length” tool on the analySIS program and was made along the long axis of the stereocilium between the tip and entry into the cuticular plate. The thickness of the stereocilia rootlets were taken from their narrowest region.

For immunogold, cochleae were excised from wild-type control mice, fixed as above using 4% paraformaldehyde in 0.1 M sodium phosphate buffer, decalcified using 5 mM EDTA containing 1% paraformaldehyde for 3 d, dehydrated, and embedded in LR-White (London resin company) as previously described [75]. After slicing in a midmodiolar plane as before, 100 nm ultrathin sections of the apical coil were cut, mounted onto 200 mesh thin bar nickel grids coated with QuickCoat glue (Agar Scientific, Stansted), and allowed to dry. To immunolabel them, the grids were placed in sequence in drops of the following solutions placed on a parafilm sheet in a moist chamber (at room temperature unless otherwise stated): 0.05 M Tris buffered saline (TBS—0.05 M Tris and 0.9% sodium chloride, pH 7.4) for 5 min, 10% goat serum (GS) in TBS containing 0.1% Tween 20 for 30 min, 1% GS-TBS containing the Eps8 primary antibody (BD Biosciences, Germany) diluted 1∶50 overnight at 4°C, 10% GS-TBS for 15 min, goat-anti mouse IgG secondary antibody conjugated to 15 nm gold particles (British Biocell, UK) diluted 1∶20 for 2 h, TBS X3, distilled H_2_O X2, and finally saturated aqueous uranyl acetate for 20 min. After washing in distilled water and drying for at least 1 h, grids were examined in a JEOL 1230 TEM operated at 100 kV. For TEM experiments we analyzed between one and two cochleae for each age/genotype tested.

## Supporting Information

Figure S1 Specificity of the Eps8 antibody. (A–C) Immunostaining of Eps8 (green) and the hair cell marker otoferlin (red) in an adult control IHC. The Eps8 protein is localized in hair cell stereocilia (arrowhead). In this and following figures, DAPI (blue) is the nuclear marker. In order to define the exact localization of Eps8, immunohistochemical images were superimposed onto the differential interference contrast (DIC) image (B, region of stereocilia enlarged in C). Dotted lines in (B) and (C) define the border between two neighboring IHCs. Note that the Eps8 protein was present at the stereociliary tips. (D–F) The Eps8 protein was not detected in IHCs from adult Eps8 knockout mice. The arrow in (F) points to the shorter stereociliary bundle compared to that of control IHCs (C). Scale bars are 10 µm, apart from those in panels (C) and (F), which are 5 µm.
(TIF)

Figure S2 Compound action potential waveform (CAP) in Eps8 mice. (A) CAP signals recorded from control (left panel) and Eps8 knockout (right panel) adult mice (P51–P57). Note that for clarity, responses in knockout mice are amplified by 5 times and shifted down. Grey dotted diagonal line guides to equivalent stimulus levels for control and knockout mice. While CAPs in control mice had low thresholds and wave amplitude progressively increased with stimulus intensity, responses in Eps8 knockout mice only became visible for stimulus levels higher than 110 dB SPL and had much smaller maximal amplitudes (25 µV in Eps8 knockouts; 450 µV in controls). (B) The thresholds for eliciting CAPs to clicks (lowest stimulus level exciting a reliable signal: left panel) or to pure tone stimulation (right panel) were significantly greater in Eps8 knockout mice (CAP pure tone 8–16 kHz: *p*<0.001). Mean values (control: black line; knockout: red lines) and individual measurements (symbols and blue lines) are shown. In Eps8 knockout mice, CAP thresholds could not be found in 3 mice and 1 mouse for click and pure tone stimulation, respectively. (C) Growth functions (Input/Output) for CAP as a function of stimulus sound pressure (dB SPL) for control (*n* = 2) and knockout (*n* = 4) mice. Note that Eps8 knockout mice showed a similar shape of CAP growth to that of controls despite the higher thresholds and smaller responses, suggesting that the difference could be accounted for by a lower sensitivity to the stimulus. In Eps8 knockout mice we could not verify whether amplitudes were able to reach control levels since the stimulus level could not be extended beyond 120 dB SPL. CAP I/O values represent peak-to-peak amplitudes.
(TIF)

Figure S3 Summating potential (SP) in Eps8 mice. (A) SP waveforms obtained from control (left) and knockout (right) mice. (B) Mean SP threshold. In one out of 4 Eps8 knockout mice SP signal could not be elicited even at 120 dB SPL stimulus level. (C) Growth function (I/O) of the summating potential (SP) at the best responsive stimulus frequency (11.3 or 16 kHz). Two and four mice were used for control and knockout mice, respectively. Note that the thresholds for eliciting SPs (reflecting the summation of the IHC depolarization signals) were significantly increased in Eps8 knockout mice (*p*<0.05, panel A and B), and the amplitudes showed a change of polarity toward the negative scale (panel C). Using electrocochleography it has been shown that the position of the electrode on the cochlear outside wall influences the polarity of the recorded signal [76]. Since in our recordings the electrode was always positioned at the round window membrane, a change of polarity could reflect a differential change in IHC excitability at a particular location along the tonotopic gradient within the cochlea or a change in the IHC receptor potential due to the high intensity stimulation used to evoke responses in Eps8 knockout mice. However, the latter possibility is unlikely since a change in polarity was not observed when a comparable, high intensity stimulus was presented to wild-type mice (only a loss of threshold and amplitude responses was seen due to a temporary hearing loss caused by the overstimulation).
(TIF)

Figure S4 Distortion product otoacoustic emissions (DPOAEs) and cochlear microphonics (CM) in Eps8 mice. The activity of OHCs was investigated by measuring DPOAEs, which originate from the mechanical nonlinearity within the cochlea, i.e. OHC electromotility [77]. In adult control animals (6–7 wk old: measurements were from 12 ears/6 mice), the DPOAE signals were found to be within the expected normal range and both the amplitudes (A: 12.5±4.7 dB SPL) and thresholds (B: 0 dB SPL at 11.3 kHz) showed that electromotile activity in control OHCs was normal. In knockout mice (4–5 wk old: measurements were from 15 ears/8 mice), the amplitude and threshold functions were very close to the technical detection limit, suggesting that OHC electromotility could have been affected. The sum of the mechano-electrical transducer currents predominantly produced by the OHCs was also investigated using cochlear microphonics (CM). The mean CM threshold (thick lines) and individual ear CM thresholds (thin lines) in control and knockout adult mice are shown in (C). CM thresholds were normal in wild-type control mice and were increased to stimulus levels close to the intrusion of the stimulus artifact (ca. 80 dB SPL) in Eps8 knockout mice (4–16 kHz, *p*<0.001). These data therefore suggest that OHC activity is likely to be compromised in Eps8 knockout animals. However, we have shown that the biophysical properties of adult Eps8 knockout OHCs, including their electromotile activity, are similar to those present in control cells (Figure 7). These findings, together with the fact that CM are absent in knockout mice, indicate that their shorter, and most likely stiffer, hair bundles are unlikely to be stimulated effectively in normal physiological conditions.
(TIF)

Figure S5 FM1-43 uptake in Eps8 control and knockout hair cells. (A) Fluorescence images from the apical coil region of control and knockout P18 cochleae taken within 20 min after a 15 s exposure to 3 µM FM1-43. Note that FM1-43 labeled OHCs (3 rows) and IHCs (1 row) in both control and knockout mice, whereas the surrounding supporting cells did not load with the dye. In agreement with previous findings [23] OHCs exhibited more intense labeling than IHCs. (B) As in panel (A) but with the DIC image superimposed. (C and D) FM1-43 labeling (3 µM for 15 s) in immature cochleae from control and knockout mice (P7). Fluorescent images were acquired using either 4 s (A and B) or 5 s (C and D) exposure time. Scale bars: 20 µm.
(TIF)

Figure S6 Main physiological and morphological differences between pre- and post-hearing IHCs from control and Eps8 knockout mice. (A and B) Schematic representation showing the membrane currents and main structural features in immature control and Eps8 knockout IHCs, respectively. Based on our experiments, the only difference in immature IHCs was the additional and much shorter stereocilia and the larger transducer current in knockout IHCs compared to controls. *I*_T_, transducer current [78],[79]; *I*_K1_, inward rectifier K^+^ current [35]; *I*_K_, delayed rectifier K^+^ current [34]; *I*_Ca_, Ca^2+^ current [32]; *I*_K(ACh)_, ACh-activated K^+^ current [36],[37]. Control and knockout IHCs also showed normal voltage responses (action potential activity [32]) and a high order exocytotic Ca^2+^ dependence (left panels [45]). Afferent (a, blue) and efferent (e, pink) fibre organization is also likely to be normal in knockout mice. (C and D) Morphological and physiological differences between control (C) and Eps8 knockout (D) adult IHCs. Adult-type K^+^ currents (*I*_K,f_, BK current [33]; *I*_K,n_, KCNQ4 current [34]) were missing in knockout IHCs that instead expressed immature-type currents. Moreover, mutant IHCs remained responsive to the efferent neurotransmitter ACh, fired action potentials instead of graded receptor potentials (left-top panels), and showed an immature exocytotic Ca^2+^ dependence (high order instead of linear: left-bottom panels). Otof and Syt IV represent the proposed Ca^2+^ sensors involved in exocytosis and vesicle replenishment, respectively [45]. Closed circles (green) at the tops of stereocilia represent Eps8. Arrows next to currents indicate the main direction of ion flow.
(TIF)

## Abbreviations

ABR: auditory brainstem response
CAP: compound action potential
CM: cochlear microphonic
DPOAE: distortion product otoacoustic emission
EP: endocochlear potential
IHCs: inner hair cells
OHCs: outer hair cells
P: postnatal day
SEM: scanning electron microscopy
SP: summating potential
TEM: transmission electron microscopy
