# Supplementary figures and images for: Eps8 Regulates Hair Bundle Length and Functional Maturation of Mammalian Auditory Hair Cells
Source: PLoS Biol. 2011 Apr 19;9(4):e1001048. doi: 10.1371/journal.pbio.1001048 (PMC3079587; doi:10.1371/journal.pbio.1001048)

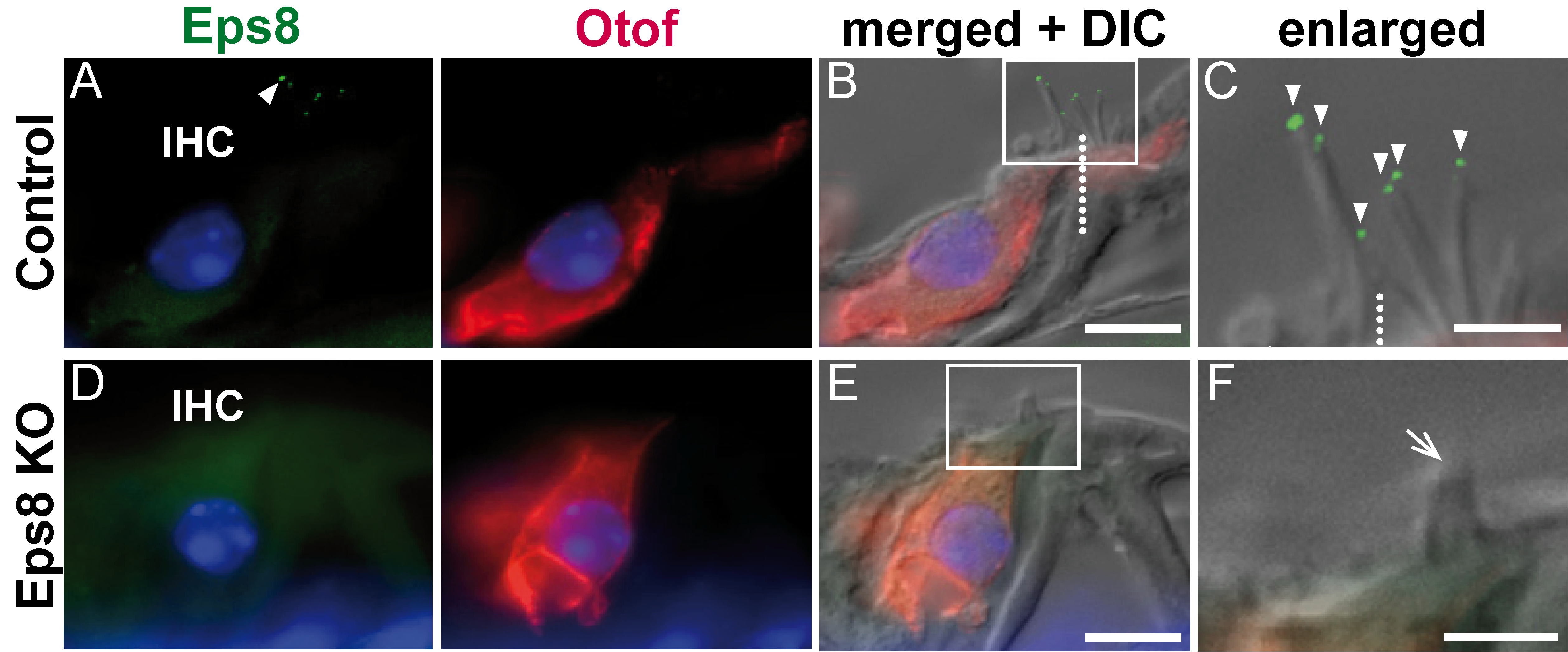

Supplement: Figure S1 — Specificity of the Eps8 antibody. (A–C) Immunostaining of Eps8 (green) and the hair cell marker otoferlin (red) in an adult control IHC. The Eps8 protein is localized in hair cell stereocilia (arrowhead). In this and following figures, DAPI (blue) is the nuclear marker. In order to define the exact localization of Eps8, immunohistochemical images were superimposed onto the differential interference contrast (DIC) image (B, region of stereocilia enlarged in C). Dotted lines in (B) and (C) define the border between two neighboring IHCs. Note that the Eps8 protein was present at the stereociliary tips. (D–F) The Eps8 protein was not detected in IHCs from adult Eps8 knockout mice. The arrow in (F) points to the shorter stereociliary bundle compared to that of control IHCs (C). Scale bars are 10 µm, apart from those in panels (C) and (F), which are 5 µm. (TIF) [file pbio.1001048.s001.tif]

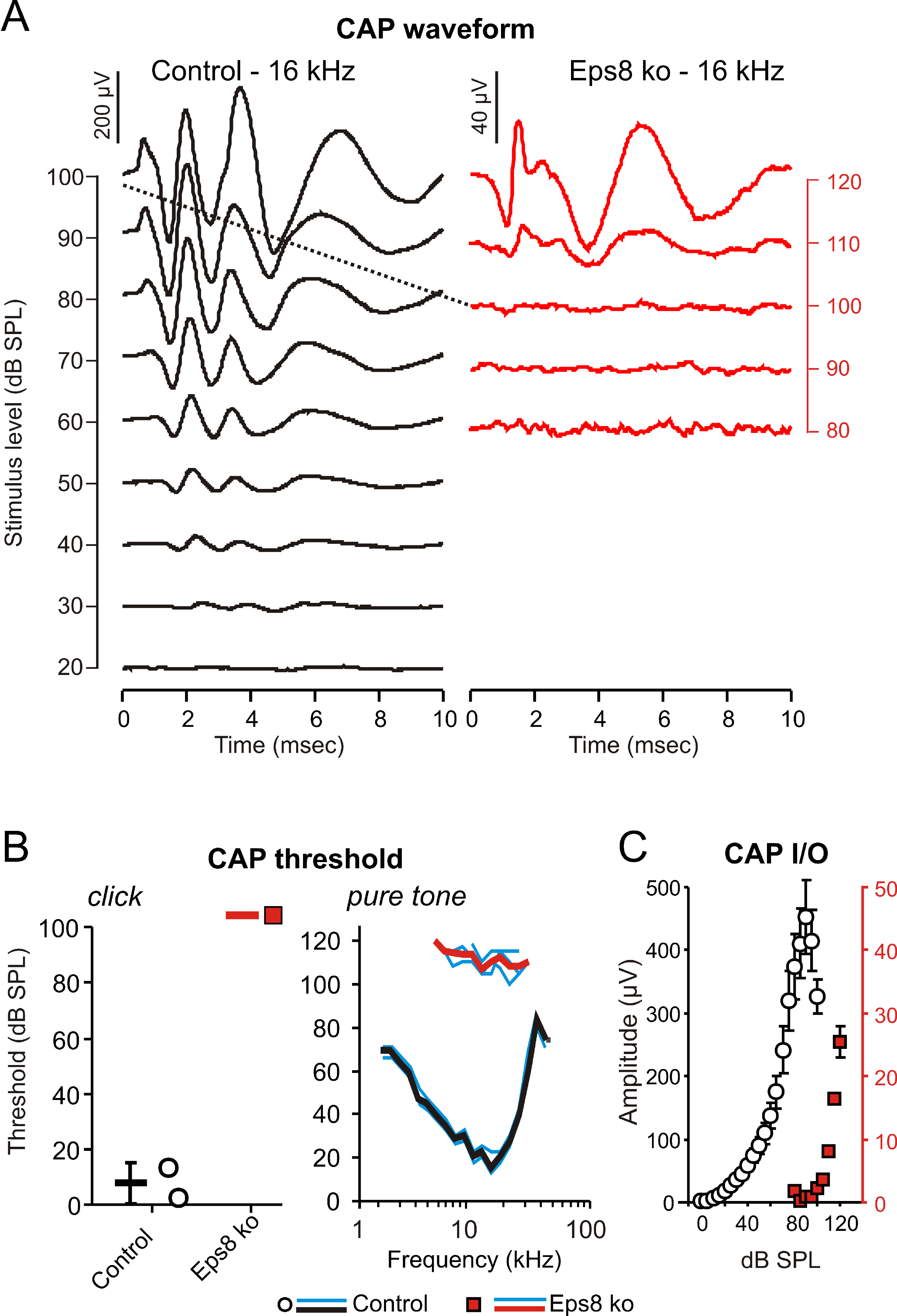

Supplement: Figure S2 — Compound action potential waveform (CAP) in Eps8 mice. (A) CAP signals recorded from control (left panel) and Eps8 knockout (right panel) adult mice (P51–P57). Note that for clarity, responses in knockout mice are amplified by 5 times and shifted down. Grey dotted diagonal line guides to equivalent stimulus levels for control and knockout mice. While CAPs in control mice had low thresholds and wave amplitude progressively increased with stimulus intensity, responses in Eps8 knockout mice only became visible for stimulus levels higher than 110 dB SPL and had much smaller maximal amplitudes (25 µV in Eps8 knockouts; 450 µV in controls). (B) The thresholds for eliciting CAPs to clicks (lowest stimulus level exciting a reliable signal: left panel) or to pure tone stimulation (right panel) were significantly greater in Eps8 knockout mice (CAP pure tone 8–16 kHz: p<0.001). Mean values (control: black line; knockout: red lines) and individual measurements (symbols and blue lines) are shown. In Eps8 knockout mice, CAP thresholds could not be found in 3 mice and 1 mouse for click and pure tone stimulation, respectively. (C) Growth functions (Input/Output) for CAP as a function of stimulus sound pressure (dB SPL) for control (n = 2) and knockout (n = 4) mice. Note that Eps8 knockout mice showed a similar shape of CAP growth to that of controls despite the higher thresholds and smaller responses, suggesting that the difference could be accounted for by a lower sensitivity to the stimulus. In Eps8 knockout mice we could not verify whether amplitudes were able to reach control levels since the stimulus level could not be extended beyond 120 dB SPL. CAP I/O values represent peak-to-peak amplitudes. (TIF) [file pbio.1001048.s002.tif]

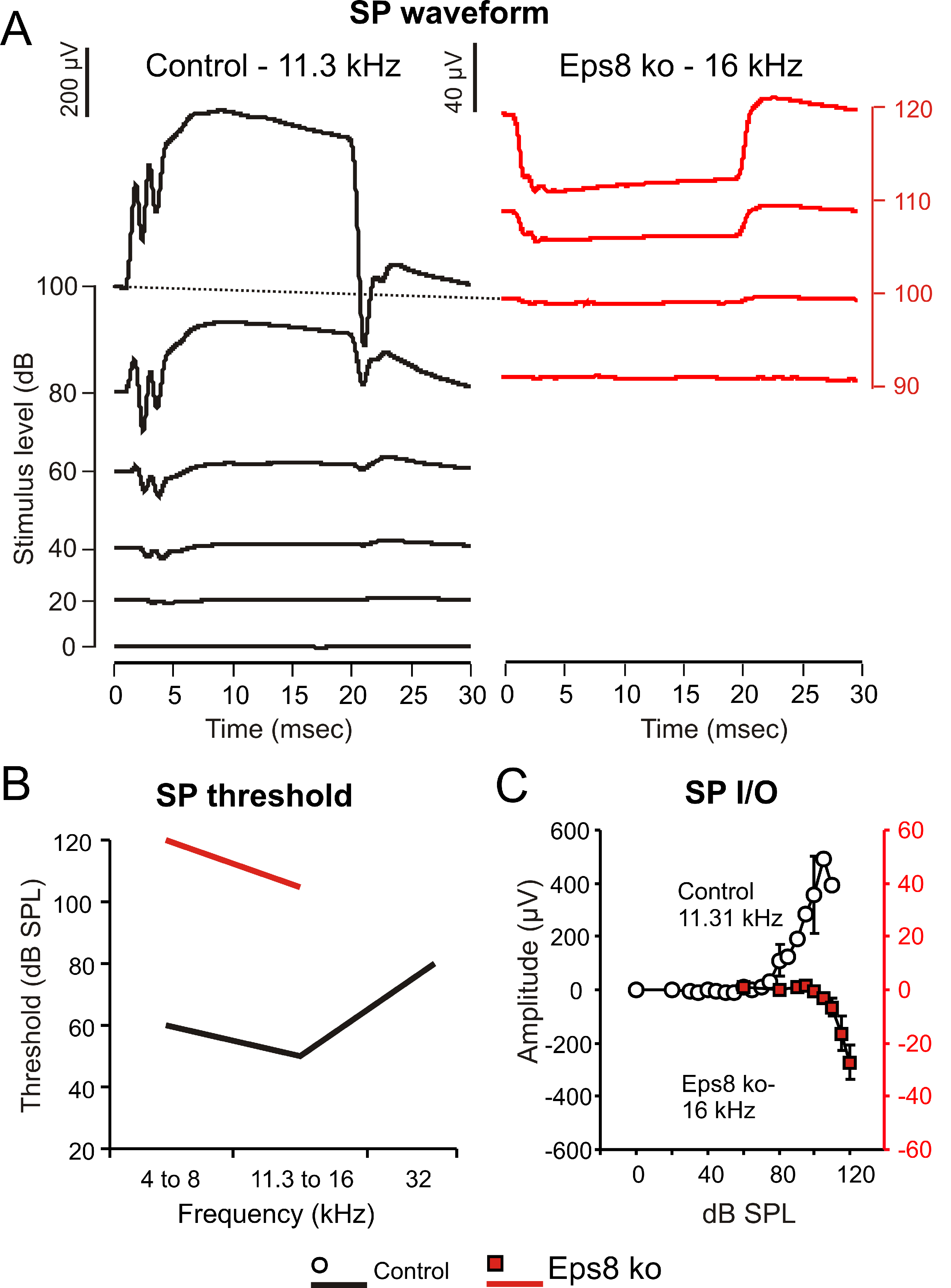

Supplement: Figure S3 — Summating potential (SP) in Eps8 mice. (A) SP waveforms obtained from control (left) and knockout (right) mice. (B) Mean SP threshold. In one out of 4 Eps8 knockout mice SP signal could not be elicited even at 120 dB SPL stimulus level. (C) Growth function (I/O) of the summating potential (SP) at the best responsive stimulus frequency (11.3 or 16 kHz). Two and four mice were used for control and knockout mice, respectively. Note that the thresholds for eliciting SPs (reflecting the summation of the IHC depolarization signals) were significantly increased in Eps8 knockout mice (p<0.05, panel A and B), and the amplitudes showed a change of polarity toward the negative scale (panel C). Using electrocochleography it has been shown that the position of the electrode on the cochlear outside wall influences the polarity of the recorded signal [76]. Since in our recordings the electrode was always positioned at the round window membrane, a change of polarity could reflect a differential change in IHC excitability at a particular location along the tonotopic gradient within the cochlea or a change in the IHC receptor potential due to the high intensity stimulation used to evoke responses in Eps8 knockout mice. However, the latter possibility is unlikely since a change in polarity was not observed when a comparable, high intensity stimulus was presented to wild-type mice (only a loss of threshold and amplitude responses was seen due to a temporary hearing loss caused by the overstimulation). (TIF) [file pbio.1001048.s003.tif]

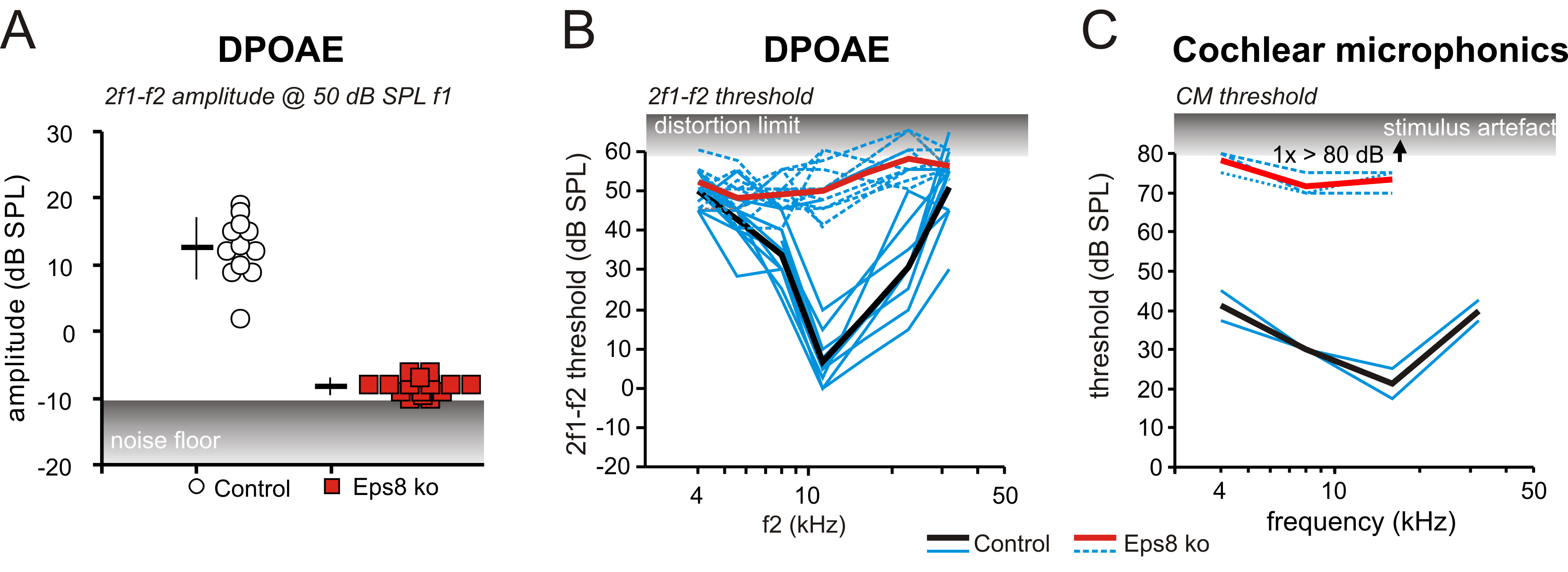

Supplement: Figure S4 — Distortion product otoacoustic emissions (DPOAEs) and cochlear microphonics (CM) in Eps8 mice. The activity of OHCs was investigated by measuring DPOAEs, which originate from the mechanical nonlinearity within the cochlea, i.e. OHC electromotility [77]. In adult control animals (6–7 wk old: measurements were from 12 ears/6 mice), the DPOAE signals were found to be within the expected normal range and both the amplitudes (A: 12.5±4.7 dB SPL) and thresholds (B: 0 dB SPL at 11.3 kHz) showed that electromotile activity in control OHCs was normal. In knockout mice (4–5 wk old: measurements were from 15 ears/8 mice), the amplitude and threshold functions were very close to the technical detection limit, suggesting that OHC electromotility could have been affected. The sum of the mechano-electrical transducer currents predominantly produced by the OHCs was also investigated using cochlear microphonics (CM). The mean CM threshold (thick lines) and individual ear CM thresholds (thin lines) in control and knockout adult mice are shown in (C). CM thresholds were normal in wild-type control mice and were increased to stimulus levels close to the intrusion of the stimulus artifact (ca. 80 dB SPL) in Eps8 knockout mice (4–16 kHz, p<0.001). These data therefore suggest that OHC activity is likely to be compromised in Eps8 knockout animals. However, we have shown that the biophysical properties of adult Eps8 knockout OHCs, including their electromotile activity, are similar to those present in control cells (Figure 7). These findings, together with the fact that CM are absent in knockout mice, indicate that their shorter, and most likely stiffer, hair bundles are unlikely to be stimulated effectively in normal physiological conditions. (TIF) [file pbio.1001048.s004.tif]

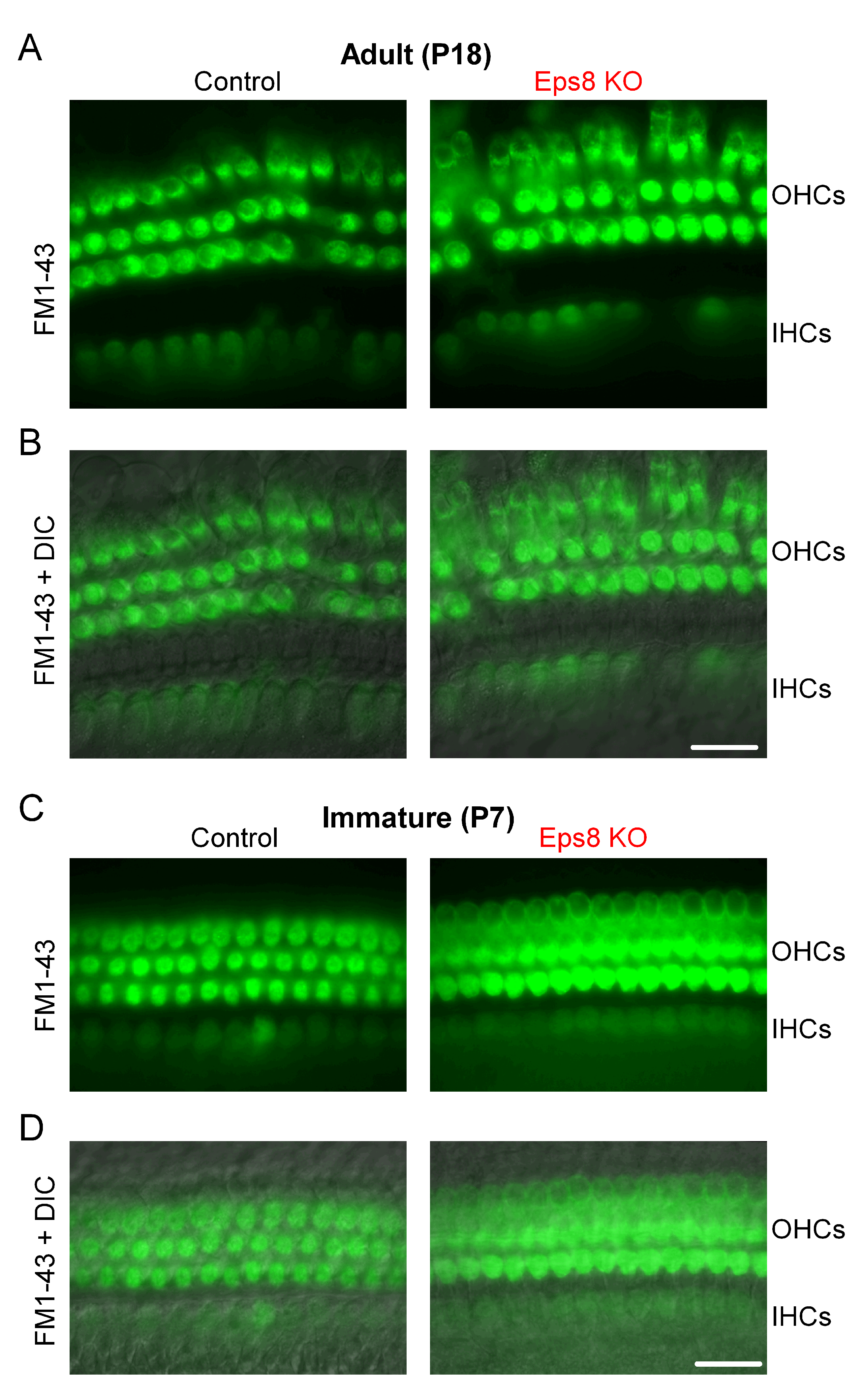

Supplement: Figure S5 — FM1-43 uptake in Eps8 control and knockout hair cells. (A) Fluorescence images from the apical coil region of control and knockout P18 cochleae taken within 20 min after a 15 s exposure to 3 µM FM1-43. Note that FM1-43 labeled OHCs (3 rows) and IHCs (1 row) in both control and knockout mice, whereas the surrounding supporting cells did not load with the dye. In agreement with previous findings [23] OHCs exhibited more intense labeling than IHCs. (B) As in panel (A) but with the DIC image superimposed. (C and D) FM1-43 labeling (3 µM for 15 s) in immature cochleae from control and knockout mice (P7). Fluorescent images were acquired using either 4 s (A and B) or 5 s (C and D) exposure time. Scale bars: 20 µm. (TIF) [file pbio.1001048.s005.tif]

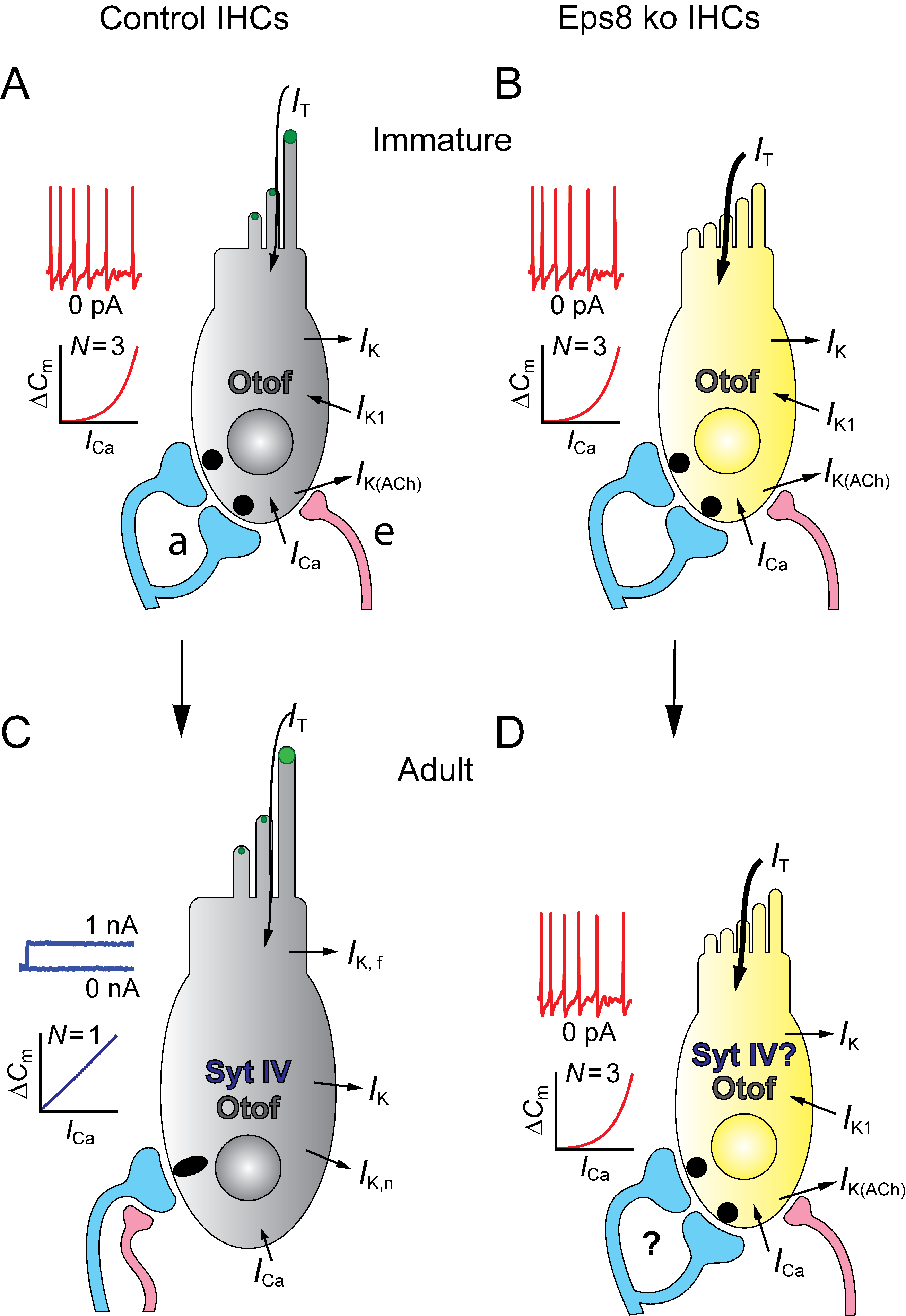

Supplement: Figure S6 — Main physiological and morphological differences between pre- and post-hearing IHCs from control and Eps8 knockout mice. (A and B) Schematic representation showing the membrane currents and main structural features in immature control and Eps8 knockout IHCs, respectively. Based on our experiments, the only difference in immature IHCs was the additional and much shorter stereocilia and the larger transducer current in knockout IHCs compared to controls. I T, transducer current [78],[79]; I K1, inward rectifier K+ current [35]; I K, delayed rectifier K+ current [34]; I Ca, Ca2+ current [32]; I K(ACh), ACh-activated K+ current [36],[37]. Control and knockout IHCs also showed normal voltage responses (action potential activity [32]) and a high order exocytotic Ca2+ dependence (left panels [45]). Afferent (a, blue) and efferent (e, pink) fibre organization is also likely to be normal in knockout mice. (C and D) Morphological and physiological differences between control (C) and Eps8 knockout (D) adult IHCs. Adult-type K+ currents (I K,f, BK current [33]; I K,n, KCNQ4 current [34]) were missing in knockout IHCs that instead expressed immature-type currents. Moreover, mutant IHCs remained responsive to the efferent neurotransmitter ACh, fired action potentials instead of graded receptor potentials (left-top panels), and showed an immature exocytotic Ca2+ dependence (high order instead of linear: left-bottom panels). Otof and Syt IV represent the proposed Ca2+ sensors involved in exocytosis and vesicle replenishment, respectively [45]. Closed circles (green) at the tops of stereocilia represent Eps8. Arrows next to currents indicate the main direction of ion flow. (TIF) [file pbio.1001048.s006.tif]
